# Kinesin-1 and mitochondrial motility control by discrimination of structurally equivalent but distinct subdomains in Ran-GTP-binding domains of Ran-binding protein 2

**DOI:** 10.1098/rsob.120183

**Published:** 2013-03

**Authors:** Hemangi Patil, Kyoung-in Cho, James Lee, Yi Yang, Andrew Orry, Paulo A. Ferreira

**Affiliations:** 1 Department of Ophthalmology, Duke University Medical Center, 2351 Erwin Road, DUEC 3802, Durham, NC 27710, USA; 2 Department of Pathology, Duke University Medical Center, 2351 Erwin Road, DUEC 3802, Durham, NC 27710, USA; 3 Molsoft, San Diego, CA 92121, USA

**Keywords:** kinesin, mitochondria, Ran-binding protein 2, ran GTPase

## Abstract

The pleckstrin homology (PH) domain is a versatile fold that mediates a variety of protein–protein and protein–phosphatidylinositol lipid interactions. The Ran-binding protein 2 (RanBP2) contains four interspersed Ran GTPase-binding domains (RBD*_n_*_= 1–4_) with close structural homology to the PH domain of Bruton's tyrosine kinase. The RBD_2_, kinesin-binding domain (KBD) and RBD_3_ comprise a tripartite domain (R_2_KR_3_) of RanBP2 that causes the unfolding, microtubule binding and biphasic activation of kinesin-1, a crucial anterograde motor of mitochondrial motility. However, the interplay between Ran GTPase and R_2_KR_3_ of RanBP2 in kinesin-1 activation and mitochondrial motility is elusive. We use structure–function, biochemical, kinetic and cell-based assays with time-lapse live-cell microscopy of over 260 000 mitochondrial-motility-related events to find mutually exclusive subdomains in RBD_2_ and RBD_3_ towards Ran GTPase binding, kinesin-1 activation and mitochondrial motility regulation. The RBD_2_ and RBD_3_ exhibit Ran-GTP-independent, subdomain and stereochemical-dependent discrimination on the biphasic kinetics of kinesin-1 activation or regulation of mitochondrial motility. Further, KBD alone and R_2_KR_3_ stimulate and suppress, respectively, multiple biophysical parameters of mitochondrial motility. The regulation of the bidirectional transport of mitochondria by either KBD or R_2_KR_3_ is highly coordinated, because their kinetic effects are accompanied always by changes in mitochondrial motile events of either transport polarity. These studies uncover novel roles in Ran GTPase-independent subdomains of RBD_2_ and RBD_3_, and KBD of RanBP2, that confer antagonizing and multi-modal mechanisms of kinesin-1 activation and regulation of mitochondrial motility. These findings open new venues towards the pharmacological harnessing of cooperative and competitive mechanisms regulating kinesins, RanBP2 or mitochondrial motility in disparate human disorders.

## 2. Introduction

Ran GTPase is a pleiotropic and master regulatory G protein switch of nucleocytoplasmic trafficking [1]. The active form of Ran GTPase, Ran-GTP, has two high-affinity targets: Ran-binding protein 1 (RanBP1) and Ran-binding protein 2 (RanBP2, also called Nup358) [2–4]. RanBP1 is an evolutionarily conserved and approximately 26 kDa single domain and cytosolic protein [2,5,6]. Conversely, RanBP2 is not evolutionarily conserved and is an approximately 358 kDa scaffold comprising four Ran GTPase-binding domains (RBD*_n_*_= 1–4_), which are interspersed between apparently unrelated structural and functional domains [7–9]. Prior studies support that RanBP1 and the RBD*_n_*_= 1–4_ of RanBP2 are structurally and biochemically alike, and present structural homology to versatile pleckstrin homology (PH) domains, such as that of the Bruton's tyrosine kinase [3,4,10–12]. In interphase cells, RanBP2 is localized among other locales at cytoplasmic filaments emanating from the nuclear pore, where it associates with nuclear import and export receptors [7,8,13,14]. Current data support that RanBP1 and the RBD*_n_*_= 1–4_ of RanBP2 in the presence of cytosolic and sumoylated RanGAP co-stimulate the GTPase activity of Ran GTPase bound to nuclear export receptors and cargoes upon exiting the nuclear pore [2,10,15–17]. This docking step is thought to be critical to trigger the allosteric destabilization and release of Ran GTPase, nuclear receptors and cargoes [4,11].

Several lines of evidence support that RanBP2 mediates multiple and apparently disparate cell-cycle- and cell-context-dependent processes in health and disease states. RanBP2 is implicated in nucleocytoplasmic transport [13,14,18–22], microtubule-based intracellular trafficking [23–27], viral infections by HIV-1 and adenovirus [27,28], nuclear envelope breakdown and mitosis [25,29–32], modulation of protein homeostasis by the ubiquitin–proteasome system [33,34], mitochondrial function and trafficking [24,35], regulation of protein–protein interactions and localizations by SUMOylation [17,36–38], negative regulation of cAMP signalling [39], and gene–environment interactions cross-talking to the regulation of glucose and lipid metabolism [35,40–42]. Although complex and not well understood, RanBP2's pleiotropic and cell-context-dependent properties reflect most likely combinatorial functions of (i) the association of distinct domains of RanBP2 with diverse functional factors, (ii) multi-functional properties associated to single domains of RanBP2, or (iii) cross-talk between adjacent domains of RanBP2 and its partners. For example, the RBD_4_ of RanBP2 was found to associate with the G protein-coupled receptor, M-opsin, an effect that is chaperoned by its adjacent cyclophilin (CY) domain. The combinatorial effect of the RBD_4_-CY domains promotes the production of functional M-opsin [43,44]. RanBP2 via its kinesin-binding domain (KBD) associates also with kinesin-1, a microtubule-based and anterograde dimeric motor [23,24]. The RBD_2_ and RBD_3_ of the tripartite domain, RBD_2_–KBD–RBD_3_ (R_2_KR_3_), of RanBP2 are critical to unfold kinesin-1, to relieve the inhibition of the C-terminal tail over the N-terminal motor domain of kinesin-1 and to enhance directly its microtubule-based motor activity upon activation by KBD of RanBP2 [45]. However, it is unknown whether the novel functions associated with the structural equivalent RBD_2_ and RBD_3_ reflect hitherto unappreciated intrinsic structural and functional properties harboured by these domains or extrinsic roles resulting from their association with Ran-GTP.

A diverse number of cargoes are reported to associate with kinesin-1 [46–48], but RanBP2 is the only native cargo known to activate directly the motor activity of a kinesin (kinesin-1) in a minimal reconstitution system [45]. Prior studies found that the KBD domain of RanBP2 associates directly with approximately 100 residues shared by the C-terminal tail and cargo-binding domain of two highly homologous kinesin-1 subtypes, KIF5B and KIF5C, but not KIF5A [23,24]. KIF5B and KIF5C are functionally redundant, and they are the primary motors mediating the outward (anterograde) transport of mitochondria [49–53], an effect that is affected by the ectopic cellular expression of KBD by mechanisms not understood [24]. Further, the R_2_KR_3_ of RanBP2 plays a critical role in the biphasic activation of kinesin-1 by binding to at least two cargo-binding sites in a multi-partite cargo-binding domain of each monomer of kinesin-1 tail [45]. However, the role(s) of the interplay between Ran GTPase and the RBD_2_ and RBD_3_ of R_2_KR_3_ in the regulation of kinesin-1 activation and their effect(s) on mitochondrial motility are lacking. This information is critical, because it will provide novel insights into multi-functional properties of RBD_2_ and RBD_3_, and the regulation of fast mitochondrial transport by kinesin-1. In addition, the regulations of kinesin-1 and mitochondrial motilities are also of high therapeutic value, because they play critical roles in a large number of disparate pathophysiologies lacking therapeutic approaches [54–59]. These unmet clinical needs are compounded further by the scarcity and lack of specificity of pharmacological agents towards kinesins. These pharmacological limitations arise from the non-selective, drug-mediated inhibition/targeting of the kinesin motor domains, which are highly conserved between members of the kinesin superfamily [60–63]. Hence, the understanding of the modulatory activities underlying intramolecular interactions between the poorly and highly conserved tail and motor domains of kinesins, respectively, intermolecular interactions between kinesin tails and their cargoes, or both, will aid significantly the development of therapeutic agents with high specificity and efficacy. Here, we present the most comprehensive kinetic characterization of mitochondrial motility carried out to date to uncover and correlate biochemical and biological Ran GTPase-independent, antagonizing and multi-modal properties of KBD and R_2_KR_3_ of RanBP2 towards kinesin-1 activation and regulation of various biophysical properties of mitochondrial motility.

## 3. Results

### 3.1. Mutual exclusive interactions of RBD_2_ and RBD_3_ of R_2_KR_3_ with Ran-GTP and kinesin-1

**Figure 1.**
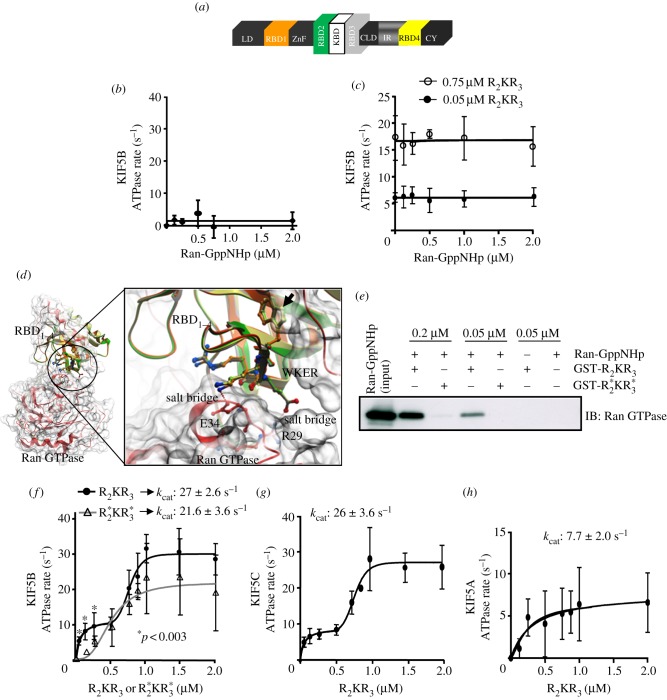
(*a*) Primary structure of RanBP2. The tripartite domain, RBD_2_–KBD–RBD_3_ (R_2_KR_3_), of RanBP2 is the focus of this study and it is shown enlarged. The RBD_2_ and RBD_3_ of R_2_KR_3_ of RanBP2 are noted with green and light grey colours. Increasing concentrations of Ran-GTP have no significant effect on the kinetics of activation of KIF5B in the (*b*) absence or (*c*) presence of different concentrations of R_2_KR_3_. There were no significant differences between R_2_KR_3_ at 0.75 and 1 μM (not shown). (*d*) Binary complex of Ran GTPase (red) with the overlay structures of RBD_*n* = 1–4_ of RanBP2 as coloured in (*a*). The RBD_2_, RBD_3_ and RBD_4_ were modelled to the RBD_1_ template structure of RanBP2. Mutations in RBD_2_ and RBD_3_ of this study comprised the replacement of the ‘W’ residue with ‘R’ in the WKER motif (arrow). The W → R mutation disrupts highlighted salt–bridge interactions of the K and E residues in the conserved WKER motif of RBDs with the oppositely charged E34 and R29 residues, respectively, of the switch-I of Ran GTPase. The distances E34–K58 and R29–E59 are 2.18 Å and 1.68 Å, respectively. (*e*) The W → R mutation in the WKER motifs of RBD_2_ and RBD_3_ of R_2_KR_3_, 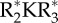, abrogates the binding of Ran-GppNHp to 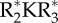. (*f*) R_2_KR_3_ promotes the biphasic kinetic activation of KIF5B that is composed by hyperbolic and cooperative/sigmoid phases. The mutant 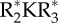 suppresses strongly and selectively the hyperbolic phase of kinesin activation (ANOVA two-way repetitive measures, *p* < 0.003) and lowers mildly, albeit not significantly, the catalytic efficiency (*k*_cat_) of KIF5B. (*g*) The biphasic kinetic activation of KIF5C by R_2_KR_3_ and its catalytic efficiency (*k*_cat_) are indistinguishable from those of KIF5B (*f*). (*h*) R_2_KR_3_ weakly stimulates KIF5A activity without biphasic kinetics of activation. Data are means of three independent experiments ± s.d. (*b*, *c*, *f*–*h*).

The R_2_KR_3_ and domains thereof of RanBP2 (figure 1*a*) affect distinct kinetic phases of KIF5B activation [45]. We examined whether Ran GTPase modulates the activation of kinesin-1 upon Ran-GTP association with the RBD_2_ and RBD_3_ of R_2_KR_3_ or these domains in R_2_KR_3_ present non-overlapping subdomains towards kinesin-1 and Ran-GTP. First, we determined the effects of increasing concentrations of Ran GTPase charged with the stable non-hydrolysable analogue of GTP, Ran-GppNHp (5′-guanosyl-[β,γ-imido]-triphospahte), on the linear rates of KIF5B activation in the absence (figure 1*b*) and presence of three concentrations of R_2_KR_3_ (0.05, 0.75 and 1 μM; figure 1*c*; see also figure 1*e*). Notably, Ran-GppNHp neither had an effect on the ATPase activity of KIF5B nor stimulated its activity (figure 1*b*). On the other hand, increasing R_2_KR_3_ concentrations promoted an increase in KIF5B activity, but its activity remained unchanged under any concentration of Ran-GppNHp tested (figure 1*c*). Hence, the RBD_2_ and RBD_3_ of R_2_KR_3_ present non-overlapping binding subdomains towards KIF5B and Ran-GppNHp.

To examine further the role of the interactions of RBD_2_ and RBD_3_ of R_2_KR_3_ with Ran-GTP and KIF5B, we took advantage of a mutation, W → R, identified serendipitously in the conserved ‘WKER’ motif of RBD_3_ of RanBP2. The atomic structure of the Ran-GppNHp-RBD_1_ complex [4] and structural modelling of RBD*_n_*_= 2–4_ of RanBP2 to RBD_1_ predict that the W → R mutation disrupts the association of RBD*_n_*_= 1–4_ with Ran-GTP (figure 1*d*). The WKER residues are 100 per cent conserved between RanBP1 of all species and RBD*_n_*_= 1–4_ of RanBP2 [4]. This motif forms electrostatic interactions with the switch I loop region (residues 29–38) of Ran-GTP, thereby preventing the intrinsic and EDTA-stimulated release of GTP from Ran-GTP [4]. In addition to destabilizing the electrostatic interactions of the WKER motif with Ran-GTP (figure 1*d*) [4], the bulky hydrophobic to hydrophilic residue change of W → R at the beginning of β2-sheet is predicted to cause the structural disruption (local unpacking) of neighbouring residues, because of the burying of a charged arginine residue (R) without a counter-charge in close proximity.

To validate experimentally these predictions, we first carried out pull-down assays of purified wild-type R_2_KR_3_ or mutant constructs thereof with the W2052R in RBD_2_ and W2349R in RBD_3_ 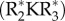 upon incubation with Ran-GppNHp. As shown in figure 1*e*, wild-type R_2_KR_3_ co-precipitated Ran-GppNHp, whereas the counterpart mutant construct, 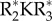, failed to do so at any concentrations tested. Then, to probe the unpacking effect of W → R in the RBD_2_ and RBD_3_ of 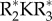 on kinesin-1 activation, we compared the activation kinetics of KIF5B in the presence of R_2_KR_3_ or 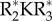. We have shown previously that R_2_KR_3_ causes the biphasic kinetics of kinesin-1 activation. This activation kinetics is characterized by an initial hyperbolic (stimulatory) phase followed by a sigmoidal (cooperative) phase owing to distinct activation of binding sites in kinesin-1 tail towards selective domains of R_2_KR_3_ [45]. By using the same kinetic activation assays of kinesin-1 reported previously [45], we found that 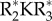 suppressed significantly the hyperbolic phase of KIF5B activation without affecting its cooperative (sigmoid) kinetic phase (figure 1*f*). Although not significant, this effect led also to a tenuous decrease in the ATPase activity of KIF5B (27 ± 2.6 versus 21.6 ± 3.6 s^−1^; figure 1*f*). Hence, these results further strengthen the notion that KIF5B presents multi-modal stimulatory activities. Further, the cooperative phase of KIF5B activation is not dependent on its initial hyperbolic activation phase, because the hyperbolic phase can be suppressed without impairing significantly the catalytic efficiency of KIF5B.

Finally, we probed the selectivity of R_2_KR_3_ towards its effects on the activation to two other kinesin-1 isoforms, KIF5C and KIF5A. KIF5B and KIF5C are orthologous kinesin-1 isoforms [50,51], which are known to associate specifically with RanBP2 [23,24], whereas KIF5A is implicated in distinct intracellular trafficking and disease processes [64,65], and it is thought not to associate with RanBP2 or domains therein [23,24]. As shown in figure 1*g*,*h*, R_2_KR_3_ had indistinguishable stimulatory biphasic and kinetic effects on KIF5C activity compared with KIF5B (figure 1*g*; compare also with figure 1*f*), but its stimulatory effect on KIF5A was weak and did not present a biphasic kinetics of activation (figure 1*h*). Hence, R_2_KR_3_ of RanBP2 is highly selective towards the highly homologous KIF5B and KIF5C isoforms.

### 3.2. Analyses of mitochondrial motility

**Figure 2.**
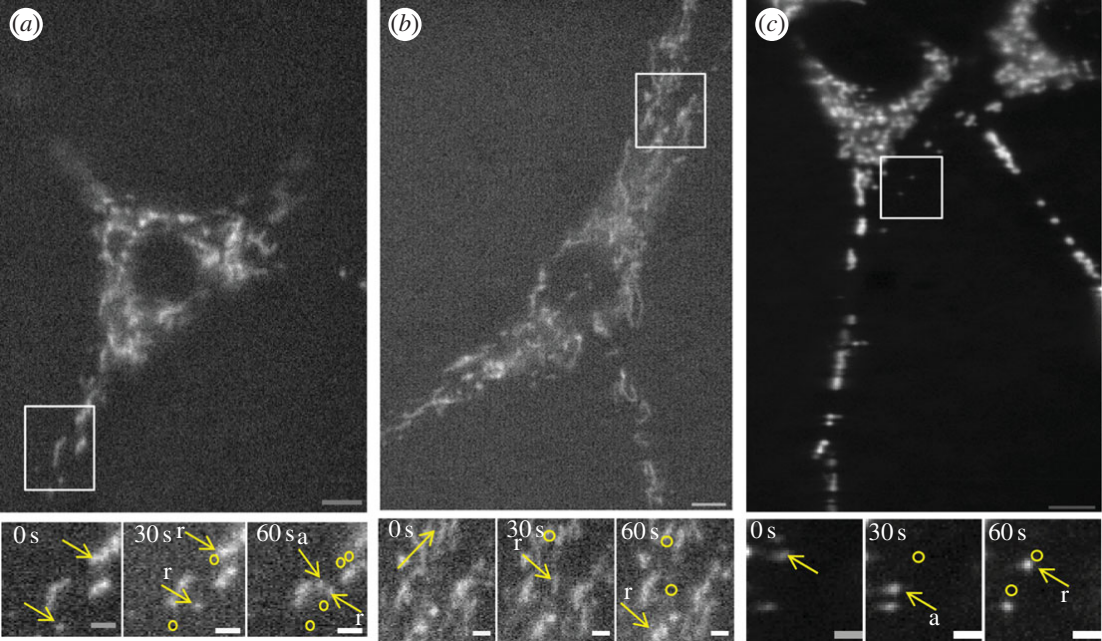
Time-lapse microscopy of mitochondrial distribution and motility of 3T3 cells transfected with (*a*) KBD after 12 h, (*b*) R_2_KR_3_ after 15 h or (*c*) non-transfected cells (NT). Lower panels are higher magnifications of still images at three different time points taken from movies of regions of interest (insets) depicted in the upper panels. Yellow arrows represent mitochondria undergoing retrograde (r) or anterograde (a) motility. Circles depict prior coordinates (positions) of mitochondria. Movies of upper and lower panel images are shown in the electronic supplementary material. Scale bars of upper and lower panels are 13 and 4 μm, respectively.

Next, we examined functional relationships between the kinetics of kinesin-1 activation in the presence of KBD or R_2_KR_3_ constructs as described herein and elsewhere [45], and the regulation of fast mitochondrial transport by kinesin-1 upon ectopic expression of such constructs of RanBP2. To this effect, we used time-lapse live cell microscopy of cultured NIH-3T3 fibroblasts expressing wild-type and mutant YFP-tagged RanBP2 mini-constructs comprising KBD or R_2_KR_3_ without and with loss-of-function mutations in KBD, RBD_2_ or RBD_3_, to dissect out their effects on multiple biophysical parameters of mitochondrial motility. This kinetic analysis excluded all fission/fusion mitochondrial events (see also §5) and its primary focus was on the detection of early kinetic changes of mitochondrial motility before downstream (secondary) subcellular phenotypes ensue from sustained mitochondrial kinetic changes as reported by prior studies (figure 2) [24]. Hence, time-course-dependent studies were carried out as soon as the expression of ectopic YFP-tagged RanBP2 mini-constructs in live cells with extended protoplasmic processes and well dispersed mitochondria were detected by epifluorescence microscopy with a high-quantum efficiency CCD camera (figure 2), but not by immunoblot analyses of 1.2 million transfected cells. These analyses mitigate potential off-target effects caused by supraphysiological concentrations of RanBP2 mini-constructs and they support the validation of concentration-dependent ligand–receptor responses of kinesin-mediated mitochondrial motility upon increasing transfection times with various RanBP2 constructs. Preliminary qualitative surveys suggested that there were overall changes in mitochondrial motility between KBD and R_2_KR_3_ of RanBP2 and mock-transfected cells (figure 2*a–c*). Thus, we pursued in depth the quantitative and stringent examination of about 20 biophysical motility-related parameters of either polarity recorded from a large pool of motile mitochondria (more than 1800) and reflecting over 260 000 motility-related events (biophysical parameter definitions are provided in electronic supplementary material). Because the average velocity of a mitochondrion transported by microtubule-based motors ranges from 0.3 to 2.0 μm s^−1^ [66–68], we recorded mitochondrial motility at two frames per second, which provides the spatial resolution above that detected by our microscopy system (approx. 240 nm). This helps to exclude noise from slow actin-based transport events of mitochondria (less than 300 nm s^−1^) [69,70] without loss of information of fast microtubule-based transport events. Although some of the tallied biophysical motility parameters are redundant, yet confirmatory to the overall significance of the analyses performed (these are described in electronic supplementary material, figures S1 and S2), each of the biophysical parameters described in table 1 offers unique mechanist insights into critical molecular mechanisms regulating kinesin-1-mediated mitochondrial motility by various domains of RanBP2.

**Table 1.** Analyses of core biophysical parameters of mitochondrial motility by KBD, R_2_KR_3_ and mutations therein. The definitions and significances of biophysical parameters are described.

biophysical parameter	definition	significance
percentage of motile events	number of motile events recorded	overall motility behaviour
absolute duration of pause between runs	duration of a non-motile event between two runs in the anterograde, retrograde or change of directions	regulation of kinesin or dynein motor activity from inactive to active states
frequency of change in direction	number of times a mitochondrion changes from anterograde to retrograde direction or vice versa	regulation of cross-talk between kinesin and dynein
motility persistency	time interval of steady movement of a mitochondrion in anterograde, retrograde or both directions	regulation of duration of motor activity of kinesin, dynein or both from active to inactive states
mean velocity	mean velocity of all motile events in the anterograde, retrograde or both directions	mean ATPase activity of kinesin, dynein or both from several runs
absolute velocity	velocity of a run (motile) event in the anterograde or retrograde direction	absolute ATPase (motor) activity of kinesin or dynein during a run; measures also net displacement resulting from the cooperation between motors (motor ensemble)

### 3.3. Kinesin-binding domain expression causes the upregulation of mitochondrial kinetics

**Figure 3.**
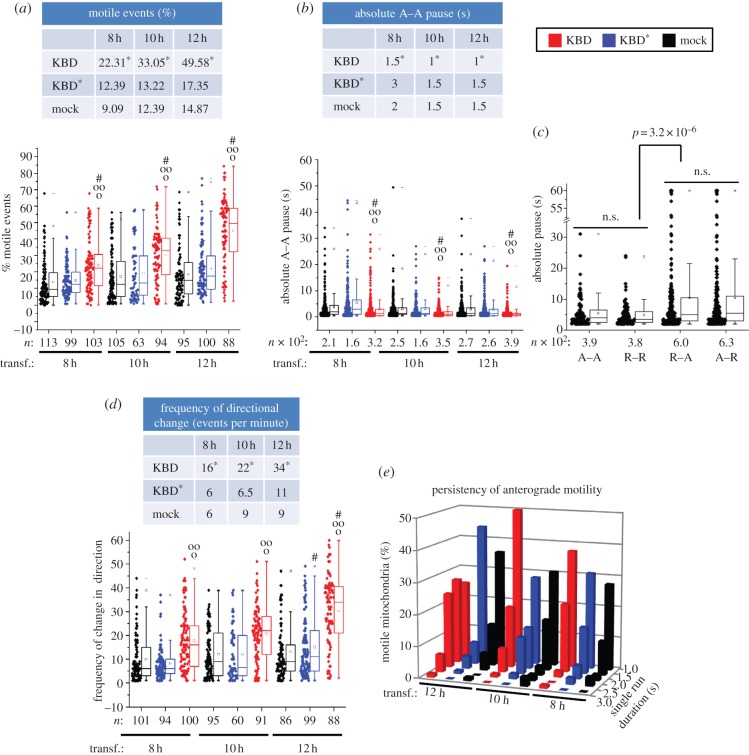
Upregulation of biophysical parameters of mitochondrial kinetics by KBD of RanBP2. Dot-box plot analyses are shown in (*a***–***d*). The tables accompanying dot-box plots represent the corresponding median values for the represented datasets. (*a*) KBD compared with KBD* and mock-transfected cells temporally increases mitochondrial motility and (*b*) decreases duration of pauses between anterograde motility events. (*c*) The duration of pauses between anterograde (A) and retrograde (R) motile events in the same direction (A–A, R–R) are smaller than those upon directional switches (R–A, A–R), and these differences are not affected by KBD or KBD^*^ (data not shown). (*d*) KBD compared with KBD* and mock-transfected cells temporally increases the frequency of directional changes of mitochondria. (*e*) Three-dimensional histogram showing that an increase in the persistency of mitochondrial anterograde motility is contingent upon KBD, but not KBD^*^ or mock-transfected cells (chi-squared contingency test, *p* < 0.001, *χ*^2^ = 39.475, Cramer's contingency coefficient *ϕ* = 0.631), and upon time of post-transfection (chi-squared contingency test, *p* < 0.001, *χ*^2^ = 38.22, Cramer's contingency coefficient *ϕ* = 0.58). Asterisks in medians denote significant differences (Mood's median test, *α* = 0.01); symbols ‘o’, ‘oo’ and hash are mitochondrial pools significantly different from non-transfected (o), KBD^*^ (oo) and other times of transfection with the same construct (hash); *n*, number of mitochondria; Mock, mock-transfected; KBD and KBD^*^ are wild-type and mutant kinesin-binding domain of RanBP2, respectively; transf., post-transfection time; n.s., not significant. (*a*–*d*) Mann–Whitney test, *p* < 0.001.

First, we began with the quantitative analyses of the effect of kinesin-binding domain (KBD) expression on mitochondrial motility. As shown in figure 3, we found that expression of KBD promotes changes in multiple biophysical parameters of mitochondrial motility. KBD produced a significant time-course-dependent upward shift (more than twofold) in overall mitochondrial motility (percentage of motile events; *p* ≤ 7.9 × 10^–4^). This effect was not observed with mock-transfected cells and a mutant KBD construct, KBD^*^, which lacks activity towards kinesin-1 binding and activation [24,45] (figure 3*a*; electronic supplementary material, table S1). Akin to this KBD-dependent effect, there was also a decrease in the fraction of stationary events observed (see electronic supplementary material, figure S1*a*). KBD, but not KBD^*^ or mock-transfected cells, also caused a temporal decrease in pauses between anterograde runs (*p* ≤ 1.2 × 10^−4^; figure 3*b*; electronic supplementary material, table S2), thus supporting an upregulation of kinesin-1 activity. Notably, we found also that the duration of the pauses between consecutive runs with directionality switches were higher than the duration of pauses between runs with the same direction (*p* ≤ 3.2 × 10^−6^; figure 3*c*; electronic supplementary material, table S2). However, these pause events were not affected between KBD, KBD^*^ or mock-transfected cells (data not shown), but they were accompanied by a KBD-dependent increase in the frequency of change in direction of motility (*p* ≤ 1.21 × 10^−5^; figure 3*d*; electronic supplementary material, table S1). These data support that KBD stimulates mitochondrial motility and the coexistence of tightly coupled and compensatory mechanisms between anterograde kinesin-1 and retrograde dynein motors in mitochondrial transport. Next, we looked at the persistence of mitochondrial motility as a functional indicator of the duration of the motor (non-stop) activity of kinesin. As shown in figure 3*e*, the persistence of mitochondrial anterograde motility is significantly contingent upon KBD as compared with KBD^*^ and mock-transfected cells at every transfection time-interval (chi-squared contingency test, *p* < 0.001, *χ*^2^ = 39.475, Cramer's contingency coefficient *ϕ* = 0.631). Likewise, the persistency of anterograde motility of mitochondria in KBD-transfected cells is significantly contingent upon hours of transfection (chi-squared contingency test, *p* < 0.001, *χ*^2^ = 38.22, Cramer's contingency coefficient *ϕ* = 0.58), with 12 h of post-transfection presenting the highest value (figure 3*e*; electronic supplementary material, table S3). Hence, the upregulation of the persistence of the motor-driven anterograde and retrograde mitochondrial transport by KBD are also tightly coupled processes.

**Figure 4.**
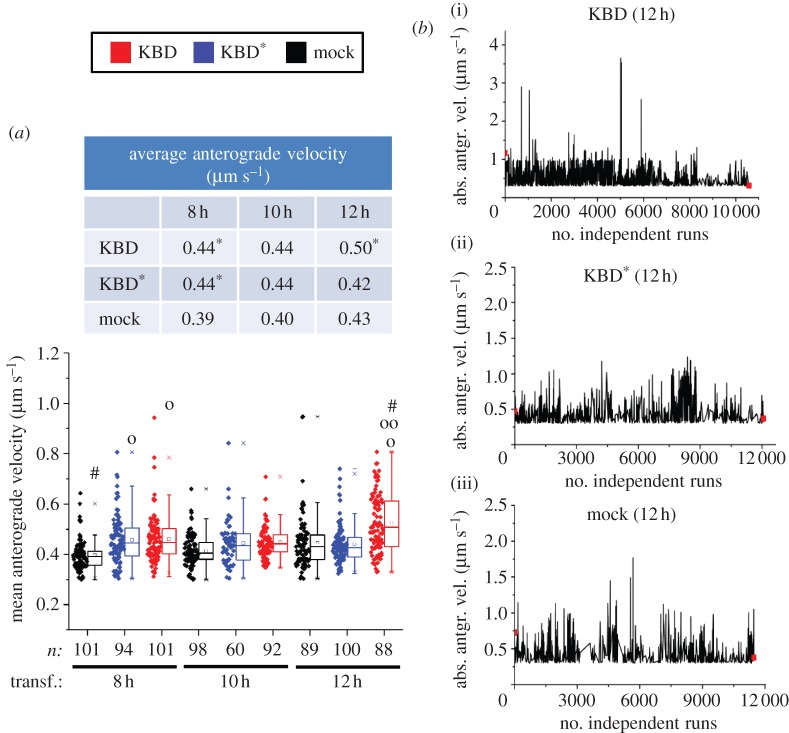
Upregulation of mitochondrial velocities by KBD of RanBP2. (*a*) Dot-box plot analyses of mean anterograde velocity of mitochondria. KBD compared with KBD^*^ and mock-transfected cells temporally increases the mean anterograde velocity of mitochondria at 12 h post-transfection. The table accompanying the dot-box plot represents the corresponding median values for the represented dataset. (*b*) Sparkline analyses of absolute velocity peaks of mitochondrial runs of (i) KBD, (ii) KBD^*^ and (iii) mock-transfected cells after 12 h of transfection show the presence of high-velocity peaks reaching 3.8 µm s^−1^ only in KBD-transfected cells. Asterisks in medians denote significant differences (Mood's median test, *α* = 0.01); symbols ‘o’, ‘oo’ and hash are mitochondrial pools significantly different from non-transfected (‘o’), KBD^*^ (‘oo’) and other times of transfection with the same construct (hash); *n*, number of mitochondria; Mock, mock-transfected; KBD and KBD^*^ are wild-type and mutant kinesin-binding domain of RanBP2, respectively; transf., post-transfection time. (*a*) Mann-Whitney test, *p* < 0.001.

Finally, we examined the mean and absolute anterograde velocities of mitochondrial motile events (figure 4*a*,*b*). The former value reflects the average velocity of all motile events recorded in 121 frames, whereas the latter is the velocity of every run (table 1). The absolute velocity correlates directly with the absolute ATPase motor activity of kinesin or the cooperation of multiple motors coupled to the same mitochondrion causing an increase of its net displacement (table 1). Notably, the mean anterograde velocity of mitochondria in KBD-transfected cells (*M* = 0.5 µm s^−1^, interquartile range, IQR = 0.43–0.61 µm s^−1^) became significantly higher only after 12 h of transfection compared with KBD^*^ (*M* = 0.42 µm s^−1^, IQR = 0.38–0.46 µm s^−1^; *p* ≤ 4.05 × 10^−6^) and mock-transfected cells (*M* = 0.43 µm s^−1^, IQR = 0.37–0.47 µm s^−1^; *p* ≤ 1.1 × 10^−7^; figure 4*a*; electronic supplementary material, table S1). Likewise, runs with high absolute velocity peaks were detected only at 12 h in the presence of KBD with the highest value recorded at 3.8 µm s^−1^ (figure 4*b*; electronic supplementary material, table S2). Thus, the time-dependent expression of KBD promotes the time-dependent upregulation of motor-driven mitochondrial transport by kinesin-1. In addition to the anterograde motility parameters described, we also analysed the effects of KBD on other complementary biophysical events (e.g. displacement), and retrograde and total (anterograde and retrograde) motility-related events (see electronic supplementary material, figure S1*b*–*l*). We observed similar outcomes in all retrograde events as for those described for the anterograde events with KBD, and these were indistinguishable between KBD^*^ and mock-transfected cells. Moreover, there were no statistical differences between any anterograde and retrograde motility-related events examined. These data further support that mitochondrial motility events of opposite polarity undergo tight mechanotransduction coupling.

### 3.4 Wild-type and Ran-GTP-binding deficient constructs of R_2_KR_3_ cause the downregulation of mitochondrial kinetics

**Figure 5.**
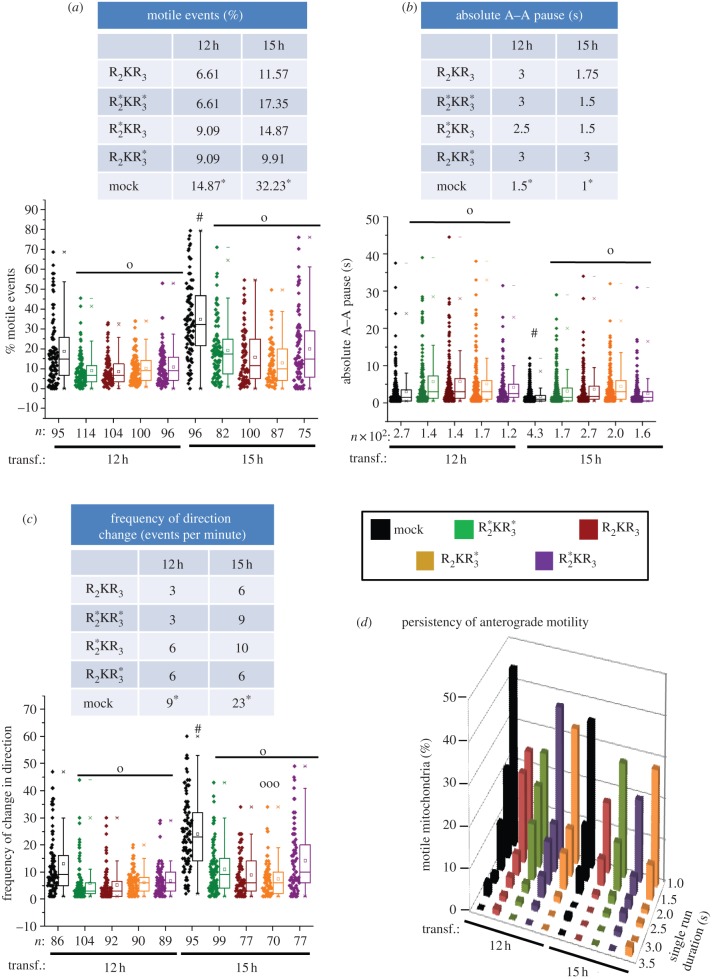
Downregulation of multiple biophysical parameters of mitochondrial motility by wild-type and mutant R_2_KR_3_ constructs of RanBP2. Dot-box plot analyses are shown in (*a***–***c*). The tables accompanying dot-box plots represent the corresponding median values for the represented datasets. Wild-type R_2_KR_3_ and constructs thereof with the W → R mutation in the WKER motif of RBD_2_ (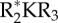), RBD_3_ (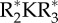) or both (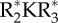) compared with mock-transfected cells cause temporally (*a*) a decrease in mitochondrial motility, (*b*) an increase in duration of pauses between anterograde motility events and (*c*) a decrease in frequency of directional changes of mitochondria. (*d*) Three-dimensional histogram showing that the persistency of anterograde motility of mitochondria is neither contingent upon any R_2_KR_3_ constructs (chi-squared contingency test, *p* > 0.05, *χ*^2^ = 20.92) nor upon the time of post-transfection (chi-squared contingency test, *p* > 0.05). Asterisks in medians denote median significant differences (Mood's median test, *α* = 0.01); symbols ‘o’, ‘ooo’ and hash are mitochondrial pools significantly different from non-transfected (o), R_2_KR_3_ constructs (ooo) and other times of transfection with the same construct (hash); *n*, number of mitochondria; Mock, mock-transfected; transf., post-transfection time. (*a*–*c*) Mann–Whitney test, *p* < 0.001.

We then examined the effects of wild-type R_2_KR_3_ and counterpart constructs with the W → R mutation in either RBD_2_ or RBD_3_ or both (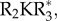 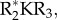 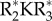) in mitochondrial motility as soon as the expression constructs were detected by CCD imaging-coupled epifluorescence microscopy. Notably, we observed a drastic reduction in mitochondrial motile events after R_2_KR_3_ transfection and irrespective of the presence or absence of the W → R mutation(s), which we showed previously to disrupt 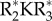 binding to Ran-GTP (*p* ≤ 3.5 × 10^−4^; figure 5*a*; electronic supplementary material, table S1). Although we observed an increase in mitochondrial motile events in R_2_KR_3_-transfected cells between 12 and 15 h of transfection, the mitochondrial motility of mock-transfected cells also showed a significant increase (figure 5*a*; electronic supplementary material, table S1). Thus, we conclude that the increase in the motile events at 15 h of post-transfection is a secondary effect not attributed to any R_2_KR_3_ construct. The effects of R_2_KR_3_ constructs on mitochondrial motility suppression were corroborated also by an increase in stationary events and a decrease in their total anterograde displacement (see electronic supplementary material, figure S2*a*,*b*) and an increase in the duration of pauses between any R_2_KR_3_ constructs and mock-transfected cells (*p* ≤ 4.7 × 10^−4^; figure 5*b*; electronic supplementary material, table S2). They were also accompanied by a significant reduction in the frequency of mitochondrial change in direction when compared with non-transfected cells at all transfection times (*p* ≤ 1.1 × 10^−5^; figure 5*c*; electronic supplementary material, table S1). Hence, these data support that R_2_KR_3_ and mutant constructs thereof suppress strongly the overall motility of mitochondria in either direction. Then, we examined the persistence of anterograde motility in the pool of motile mitochondria. There were no significant differences in motility persistence among any R_2_KR_3_ constructs (chi-squared contingency test, *p* > 0.05, *χ*^2^ = 20.92), nor did any R_2_KR_3_ constructs show anterograde motility persistence contingent upon the time of transfection (chi-squared contingency test, *p* > 0.05; figure 5*d*; electronic supplementary material, table S3). Likewise, similar observations were found for persistence in retrograde motility (see electronic supplementary material, figure S2*i*). However, we found that there was a reduction in the persistence of total mitochondrial motility, an effect that was contingent upon transfection with any R_2_KR_3_ construct (chi-squared contingency test, *p* < 0.001, *χ*^2^ = 63.49, Cramer's contingency coefficient *ϕ* = 0.32; electronic supplementary material, figure S2*j*). Hence, R_2_KR_3_ and mutant constructs thereof promote the downregulation of motor-driven mitochondrial transport.

**Figure 6.**
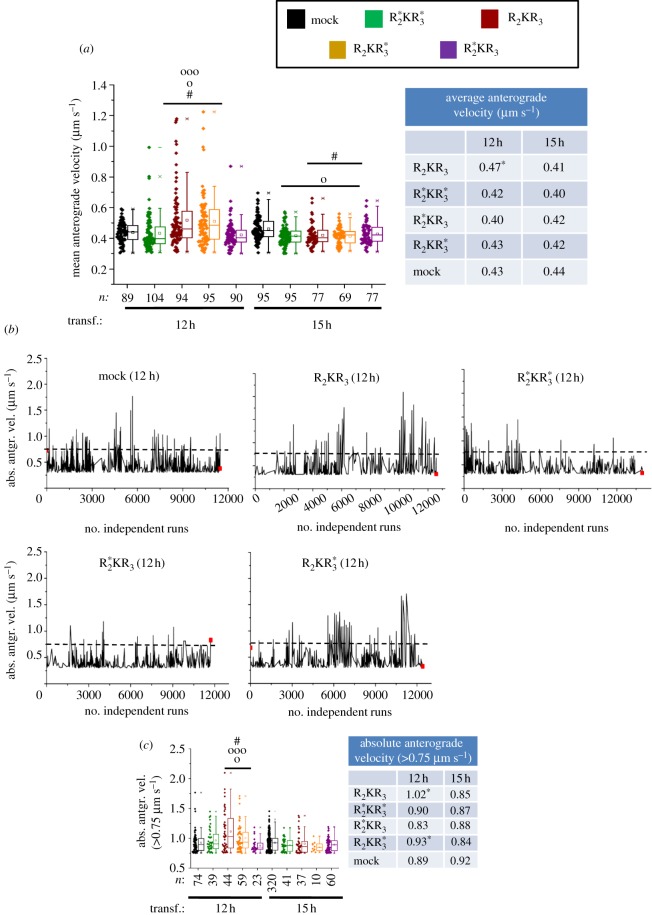
Non-equivalent and stereochemical (position)-dependent effects between RBD_2_ and RBD_3_ of R_2_KR_3_ in the regulation of kinesin-1-mediated mitochondrial velocity. (*a*) The wild-type R_2_KR_3_ and mutant R_2_KR^*^_3_ constructs cause temporally selective increases of the mean anterograde velocities of mitochondria at 12 h post-transfection (Mann–Whitney *U-*test, *p* < 0.001). Note that there is a stereochemical (position-dependent) discrimination selectively by constructs with the W2052R mutation in RBD_2_ that suppress the transient effect of the increased average anterograde velocity of mitochondria observed with other constructs at 12 h. (*b*) Sparkline analyses of absolute velocity peaks of mitochondrial runs of R_2_KR_3_, 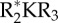, 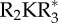 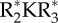 and mock-transfected cells after 12 h of transfection show the presence of velocity peaks ≥0.75 µm s^−1^ (above dashed line) mostly in R_2_KR_3_- and 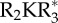-transfected cells. (*c*) The wild-type R_2_KR_3_ and mutant 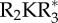 constructs cause temporally selective increases of the absolute anterograde velocities of a pool of mitochondria with velocities higher than 0.75 µm s^−1^ at 12 h post-transfection (Mann–Whitney *U*-test, *p* < 0.001). Dot-box plot analyses are shown in (*a*) and (*c*). The tables accompanying dot-box plots represent the corresponding median values for the represented datasets. Asterisks in medians denote median significant differences (Mood's median test, α = 0.01); symbols ‘o’, ‘ooo’ and hash are mitochondrial pools significantly different from non-transfected (o), R_2_KR_3_ constructs (ooo) and other times of transfection with the same construct (hash); *n*, number of mitochondria; mock, mock-transfected; transf., post-transfection time. (*a*,*c*) Mann–Whitney *U-*test, *p* < 0.001.

Finally, we examined the effect of the R_2_KR_3_ constructs on the average anterograde velocities of motile events (figure 6*a*). Mitochondria of R_2_KR_3_- and 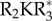-transfected cells showed significantly higher average anterograde velocity (R_2_KR_3_: *M* = 0.47 µm s^−1^, IQR = 0.40–0.59 µm s^−1^; 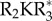: *M* = 0.43 µm s^−1^, IQR = 0.38–0.53 µm s^−1^) than mock-transfected cells at 12 but not 15 h after transfection (*p* ≤ 1.2 × 10^−5^; figure 6*a*). Notably, there was stereochemical (position dependent) discrimination between the equivalent W2052R and W2349R mutations in the structurally equivalent RBD_2_ and RBD_3_ domains, respectively, because only constructs with the W2052R mutation in RBD_2_ suppress the transient effect of the increased average anterograde velocity of mitochondria observed with other constructs at 12 h (figure 6*a*). Finally, we observed similar outcomes in all retrograde events as for those described for the anterograde events and on other complementary biophysical events with R_2_KR_3_ constructs (see electronic supplementary material, figure S2*c*–*h*,*k*–*l*).

### 3.5. Stereochemical control of mitochondrial kinetics by R_2_KR_3_

Although there were significant differences in the average anterograde velocity of mitochondria in the presence of R_2_KR_3_ or 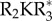 at 12 h post-transfection compared with  R^*^_2_KR^*^_3_ and mock-transfected cells, we observed no significant differences in their absolute anterograde run velocities (data not shown). Hence, to understand the apparent discrepancy between the average and absolute velocities, we examined sparkline plots that show the absolute velocity of every individual run. Notably, we observed an increased number of independent runs with high velocities when R_2_KR_3_ constructs contained the wild-type RBD_2_ domain (figure 6*b*). Hence, we hypothesized that the high frequency of lower-velocity runs masked less frequent high-velocity runs. To test this hypothesis, we divided the absolute run velocities in two independent populations (those with velocities of less than 0.75 and greater than or equal to 0.75 µm s^−1^) and analysed the two groups for statistical differences. Indeed, the dot-box plot analysis of absolute velocities greater than or equal to 0.75 µm s^−1^ showed significant increases in the run velocities of any R_2_KR_3_ construct containing the wild-type RBD_2_ (R_2_KR_3_, 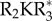), but not the mutant RBD_2_ (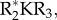 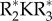) or with mock-transfected cells (figure 6*c*; electronic supplementary material, table S2). Collectively, these data support transient, non-equivalent, and stereochemical (position)-dependent functional roles of structurally equivalent RBD_2_ and RBD_3_ of RanBP2 in the regulation of kinesin-1-mediated mitochondrial motility.

### 3.6. Distinct functional subdomains are likely to be present across other Ran GTPase-binding domains of RanBP2

**Figure 7.**
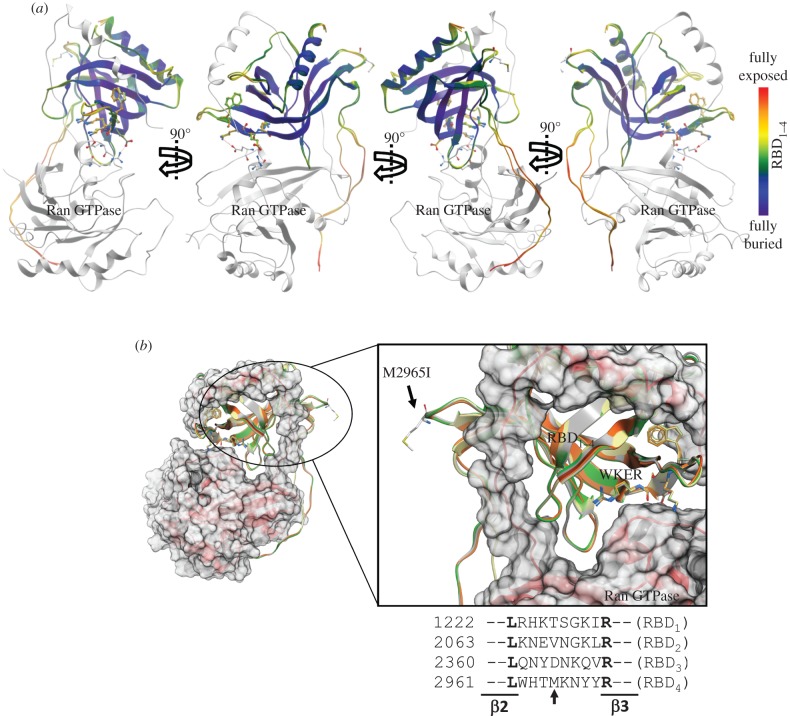
(*a*) 90°C degree views of the complex of Ran GTPase (grey ribbon) with the overlay structures of RBD*_n_*_= 1–4_ (multi-coloured) of RanBP2. The RBD*_n_*_= 2–4_ were modelled to the RBD_1_ template structure of RanBP2. The ribbon representation is coloured according to solvent accessibility, highlighting the solvent-exposed sites/domains of RBD*_n_*_= 1–4_, such as the C-terminal α_1_-helix, which may mediate the Ran-GTP-independent interaction with kinesin-1. (*b*) Binary complex of Ran GTPase (red ribbon) with the overlay structures of RBD*_n_*_= 1–4_ of RanBP2 as coloured in figure 1*d* and highlighting the solvent-exposed residue and mutation, M2965I, in the RBD_4_ of RanBP2. M2965 is displayed in stick and labelled. Alignment of the poorly conserved primary sequence of the loop between the β2 and β3 sheets of RBD*_n_*_= 1–4_ of RanBP2 is also shown. The non-conserved M2965 residue in RBD_4_ is marked by the arrow.

The data presented so far strongly support that the RBD_2_ and RBD_3_ of RanBP2 harbour mutually exclusive structural and functional subdomains towards Ran-GTP and kinesin-1. These data are also in concordance with our prior studies, where RanBP2 was found to co-associate with KIF5B and KIF5C and Ran GTPase in brain and retinal extracts [23]. Prior structural studies have shown that RBD*_n_*_= 1–4_ present a PH domain fold, which is a highly versatile fold for protein–protein and protein–phosphatidylinositol lipid interactions [4,12]. Further, structural studies on binary complexes of RBD_1_-Ran-GTP or RanBP1 and RanBP3 within other structural ensembles support the presence of structural subdomains across all RBD*_n_*_= 1–4_ that are exposed to solvent and thus free to participate in interactions with other partners (figure 7*a*) [4,11,71]. This notion is also supported by the findings that the RBD_2_ and RBD_3_, and RBD_4_, of RanBP2 interact directly with distinct partners, as shown by this and other studies [43–45]. Hence, we searched databases for pathological mutations reported in any RBD*_n_*_= 1–4_ of RanBP2 that would further strengthen the notion of the presence of multi-functional and Ran GTPase-independent activities linked to novel subdomains of RBD*_n_*_= 1–4_. We found a human mutation in the RBD_4_ of RanBP2, M2965I, which is reported to occur in 83 per cent of pancreatic cancers (figure 7*b*) [72]. Molecular modelling and overlay structures of all four RBD*_n_*_= 1–4_ of RanBP2 clearly show that the non-conserved M2965 residue is fully exposed to the solvent in a poorly conserved structural loop between the β2 and β3 sheets of RBD_4_, and away from the W → R substitution in the conserved ‘WKER’ motif (figure 7*b*) [4]. This clearly shows that the non-conserved M2965 residue does not participate in the interaction with Ran-GTP.

## 4. Discussion

These studies uncover the presence of novel subdomains in RBD_2_ and RBD_3_ of RanBP2 that are critical to kinesin-1 activation, and present non-overlapping structural and functional discrimination between Ran-GTP and kinesin-1 association. In addition, our studies uncover several kinetic properties of regulation of kinesin-1 activation, and establish direct corollaries between biochemical outcomes of single-molecule kinesin activities and the regulation of kinesin-1-mediated mitochondrial motility by KBD, R_2_KR_3_ or subdomains thereof of RanBP2. Several important conclusions can be drawn from these studies.

First, the RBD_2_ and RBD_3_ of RanBP2 present Ran-GTP-independent functions in kinesin-1 activation. The RBD_2_ and RBD_3_ present mutually exclusive interaction sites towards Ran-GTP and kinesin-1 that confer multi-functional properties to these domains. Further, we show that it is possible to dissect kinetically apart the sigmoid (cooperative) and hyperbolic phases of kinesin-1 activation by suppressing the latter upon introduction of the structurally equivalent mutation, W → R, in the conserved ‘WKER’ motifs of RBD_2_ and RBD_3_ of R_2_KR_3_. Thus, in addition to impairing Ran-GTP association to the RBDs, the W → R substitution also affects a nearby kinesin-interacting subdomain. These observations support that the hyperbolic and sigmoid (cooperative) phases of kinesin-1 activation are not fully interdependent, because the catalytic efficiencies of kinesin-1 upon R_2_KR_3_ and 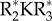 stimulations are comparable. Thus, kinesin-1 presents a multi-modal *modus operandi* by which the initial hyperbolic phase of kinesin motor activity can be bypassed. This activation phase of kinesin was thought previously to be required to ‘jump start’ the motor activity of kinesin-1 [45]. Further, these data strengthen the findings supporting the presence of two tightly conformational coupled sites for the binding of RBD_2_ and RBD_3_, and KBD, in R_2_KR_3_ to kinesin-1 [45]. Future structural studies with ternary or binary complexes of Ran-GTP-R_2_KR_3_-KIF5B/KIF5C or R_2_KR_3_-KIF5B/KIF5C should provide additional insights into the mechanisms governing the interactions of R_2_KR_3_ with Ran-GTP and KIF5B/KIF5C.

**Figure 8.**
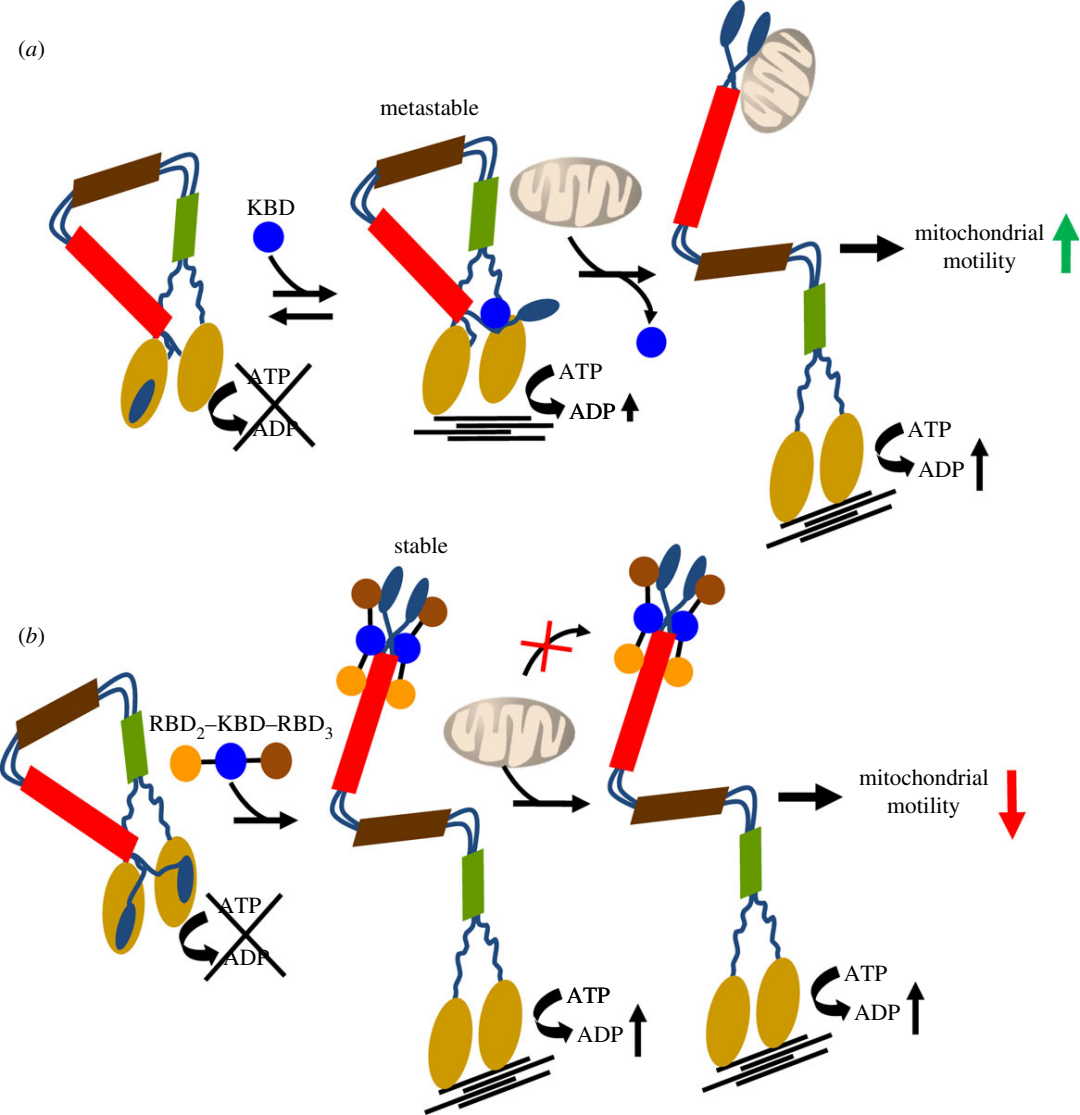
Schematic model of the regulatory effects of KBD and R_2_KR_3_ domains of RanBP2 in kinesin-1-mediated mitochondrial motility. (*a*) KBD associates weakly with the tail of kinesin-1 and produces a metastable form of kinesin-1 with low intrinsic ATPase motor activity. The metastable kinesin-1 is characterized by (i) the partial inhibition of the tail over the motor domain of kinesin and (ii) exposure of its multi-partite cargo-binding sites upon partial occupancy of the multi-partite cargo-binding domain by KBD. This facilitates the interaction between kinesin and the mitochondrion. The outcome of these effects is a kinesin-1-mediated increase in mitochondrial motility by KBD. Further, a significant rise in the number of metastable kinesins is likely to promote also an increase in the number of kinesins per mitochondrion. This causes an increase in the net displacement of the mitochondrion and thus of its velocity due to cooperation between the kinesin motors (not depicted in the model). (*b*) By contrast, R_2_KR_3_ of RanBP2 associates strongly and stably with all cargo-binding sites in the tail of kinesin-1. The full occupancy of the multi-partite cargo-binding domain in the tail of kinesin-1 by R_2_KR_3_ blocks the interaction of kinesin-1 with endogenous cargos (e.g. mitochondria), while promoting the formation of a ‘cargoless’ and active kinesin-1. Hence, the inhibition of the interactions between kinesin-1 and mitochondria by R_2_KR_3_ results in an overall reduction of kinesin-1-mediated mitochondrial motility.

Second, the data reveal two sharply contrasting biological effects of KBD alone and the tripartite domain, R_2_KR_3_, on kinesin-1-mediated mitochondrial motility. KBD promotes mitochondrial motility, whereas R_2_KR_3_ strongly suppresses such activity regardless of the mutations inhibiting Ran GTPase association to the RBD_2_ and RBD_3_ of R_2_KR_3_. These effects probably reflect distinct biochemical activities linked to KBD alone, which jump-starts kinesin activation (hyperbolic phase), and those linked to the domains RBD_2_ and RBD_3_ of R_2_KR_3_, that boost the motor activity of kinesin-1 (cooperative phase) [45]. The results support a model whereby KBD expression leads to the partial and weak occupancy of a cargo-binding site in the multi-partite cargo-binding domain of the tail of kinesin-1 [45]. This leads to the generation of a metastable and partially active form of kinesin-1. We propose that this metastable form of kinesin-1 is highly receptive to engage with its endogenous cargo (mitochondrion) and thus promote an increase in mitochondrial motility as observed upon KBD expression (figure 8*a*). By contrast, R_2_KR_3_ strongly and stably associates with kinesin-1, an effect that reflects its maximal activation of kinesin-1 *in vitro* (figure 1) [45]. However, the stable association of R_2_KR_3_ with kinesin-1 causes its full activation by the complete occupancy of all cargo-binding sites in the multi-partite cargo-binding domain of the tail of kinesin-1 by R_2_KR_3_ [45]. The outcome of this effect is the suppression of kinesin-1 engagement with its endogenous cargo (e.g. mitochondrion) *in vivo*, and thus a sharp decline in mitochondrial motility observed upon R_2_KR_3_ expression (figure 8*b*).

Third, it was revealing that there were temporal and contrasting effects of KBD alone and R_2_KR_3_ on the average and absolute velocities of mitochondria. The latency of the KBD effect in the time-dependent upregulation of mitochondrial velocities (e.g. 8–10 versus 12 h) suggests that such an effect is probably caused by the time-dependent increase in KBD expression, which in turn promotes kinesin-1-mediated mitochondrial motility. The low transfection efficiency of the cells with the RanBP2 mini-constructs prevented us from probing this issue further. However, our data strongly support the notion that the increased pool of metastable kinesin-1 produced by an increase in KBD expression promotes the coupling of several kinesin molecules to a mitochondrion, an effect that secondarily leads to an increase in the net displacement of kinesin per molecule of ATP hydrolysed without affecting its ATP turnover rate. Such a mechanism is in line with other studies proposing that the mechanical coupling of a kinesin motor ensemble comprising multiple kinesin motors attached to the same cargo results in an increase in cooperation and net displacement of the kinesin-1 ensemble [73–76]. This mechanism also explains apparent discrepancies of absolute velocities observed between single-molecule and organelle transport studies [77–79]. However, the effect of the copy number of motors engaged with a cargo on its travel velocities is challenged by studies of the transport of lipid droplets by kinesin-1 [80]. The reasons for these apparent discrepancies are unclear, but they may arise from the nature of cargoes, higher regulatory mechanisms on kinesin-1, or both [80,81].

Fourth, the initial but temporary increase in mitochondrial velocity by R_2_KR_3_ or 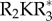, but not 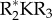 or 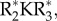 of the pool of mitochondria remaining still motile, supports a stereochemical (position-dependent) effect between the structurally equivalent RBD_2_ and RBD_3_ in the modulation of mitochondrial velocity *in vivo*. This observation suggests that the parallel or anti-parallel interaction of the coiled-coil structure (e.g. heptad leucine repeats) of KBD with the coiled-coil tail domain of kinesin-1 [24] determines non-equivalent and position-dependent effects between RBD_2_ and RBD_3_ in the regulation of kinesin-1-mediated mitochondrial velocity. Further, the position-dependent regulatory effect of RBD_2_ on mitochondrial velocity may be affected also by competitive mechanisms between RBD_2_ and other endogenous adaptors thought to couple kinesin-1 to the mitochondrion or regulate kinesin-1 stiffness [74,77]. For example, the multi-partite cargo-binding sites in kinesin-1 tail towards R_2_K domains of R_2_KR_3_ compared with KR_3_ overlap more with its kinesin light chain (KLC)-binding site [24,82], a kinesin-1 adaptor known to associate with mitochondria [83]. Other mammalian factors linking the mitochondrion directly to kinesin-1, such as Miro-1, may compete also with R_2_KR_3_ of RanBP2 or have a spatially constrained role in selective subcellular compartments, because in dendrites, Miro-1 regulates the balance between the motile and stationary phases of mitochondria, but apparently without affecting the mitochondrial average velocity [84].

Fifth, our data add to reports indicating that a tight mechanotransduction coupling mechanism operates between kinesin and dynein motors, because impairments in anterograde transport produce similar impairments in retrograde transport of mitochondria [78,85–87]. Indeed, we observed that every transport parameter recorded in one direction was accompanied by changes of opposite vectorial polarity. The notion of a tight mechanotransduction coupling mechanism between two distinct motors gains further strength with the finding that KBD of RanBP2 also binds directly to the C-terminal cargo-binding domain of the mammalian bicaudal D2 (BICD2) [25]. BICD2 acts as a dual adaptor for dynein and kinesin-1, and the competitive or cooperative interplay between these antagonizing motors, BICD2 and RanBP2, regulates the attachment of centrosomes to the nuclear envelope prior to mitotic entry [25], a mechanical checkpoint critical for the motor-driven breakdown of the nuclear envelope during late G2/early prophase [29,30]. Interestingly, other disparate mechanotransduction roles are emerging between RanBP2 and kinesin-1. For example, the activation of kinesin-1 by RanBP2 mediates adenoviral infection by promoting viral capsid disruption and uncoating at the nuclear pore and outward dispersion of capsid and selective nucleoporins, such as RanBP2 [27]. Collectively, these data support that RanBP2 mediates the tight coordination of pleiotropic motor and energy-driven trafficking processes at distinct cell cycles and between various cell types [24,25,27,88]. Future studies on the physiological effects of RBD_2_, RBD_3_ and KBD of RanBP2 with animal models should help to tease out the multi-functional activities associated with the interplay between kinesin-1, Ran GTPase and RanBP2 in mitochondrial motility and other transport events.

Finally, our studies uncover that the temporal pauses between mitochondrial runs switching directions are larger than the temporal pauses between runs with similar directions, but the temporal changes between runs switching direction are not affected by any constructs of RanBP2 used. These data support that other mechanisms independent of the constructs used control temporal switches in directionality between motors. In this regard, other factors, such as regulation of phosphatidylinositol (4,5) biphosphate (PtdIns(4,5)P2) signalling in the mitochondria or domains with PtdIns(4,5)P2-binding motifs (pleckstrin homologous), are strong candidates to modulate changes in mitochondrial directionality without impairing the overall levels of mitochondrial motility [89].

In summary, this study uncovers critical Ran-GTP-independent mechanisms modulating biochemical and antagonizing biophysical parameters of fast mitochondrial motility between kinesin-1 and KBD, R_2_KR_3_ and subdomains thereof of RanBP2. On the other hand, motor-independent and disparate functions may be associated also with Ran-GTP-independent subdomains of other Ran GTPase-binding domains of RanBP2, such RBD_4_, which is known to associate with M-opsin, and where the mutation, M2965I, in a non-conserved subdomain, is found in pancreatic cancers (figure 7*b*) [72]. The role of this mutation in pancreatic function remains unknown. However, it is noteworthy that insufficiency of *Ranbp2* is linked to glucose intolerance [35] and metabolic mitochondrial dysfunction [42], whereas kinesin-1 mediates glucose-dependent exocytosis of insulin-containing dense core secretory vesicles in pancreatic β-cells [58,90–92] and transport of glucose transport protein, GLUT4, in adipocytes [93]. It is possible that the metabolic deregulation of these kinesin-1 and RanBP2 activities contributes to the transformation of pancreatic cells. Regardless, the data presented herein open new venues to harness therapeutically multiple motile mechanisms underpinning disparate biological processes in the treatment of a variety of clinical manifestations triggered by the misregulation of microtubule-based motors, RanBP2, mitochondrial motility, or a combination of these.

## 5. Material and methods

### 5.1. Protein purification, kinetic analysis of kinesin-1 isoforms and pull-down assays

Full-length human KIF5A, KIF5B, KIF5C and R_2_KR_3_ (RBD_2_–KBD–RBD_3_), and mutants thereof, were expressed and purified as described elsewhere [45]. Purified recombinant Ran GTPase charged with GppNHp (5′-guanosyl-[β,γ-imido]-triphospahte) was kindly provided by Dr Alfred Wittinghofer. KIF5A, KIF5B, KIF5C were used at 2.5 nM in the kinetic assays. The kinetics of activation (turnover rate,  per second) of KIF5A, KIF5B, KIF5C by R_2_KR_3_ or mutant constructs thereof were performed by measuring the amount of P_i_ released with CytoPhos reagent (Cytoskeleton, Inc., Denver, CO) exactly as described previously [45]. Kinetic values were obtained by nonlinear fitting of data using GraphPad (GraphPad Software). For pull-down assays, GST-R_2_KR_3_ and 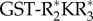 were incubated with 50 per cent slurry of glutathione-*S*1-sepharose 4B (GE) equilibrated with incubation buffer (150 mM NaCl_2_, 20 mM Tris, 1 mM EDTA, 0.4% NP40, pH 8.0) for 1 h at 24°C, washed three times followed by incubation in the same buffer with Ran-GppNHp for 2 h at 4°C. Sepharose beads were washed three times, and co-precipitates were eluted with Laemmli buffer and resolved by SDS–PAGE. Immunoblots were probed with anti-Ran antibody (1 : 100; Covance, Chantilly, VA).

### 5.2. Homology modelling of RBD_*n*  =2–4_ of RanBP2

The stochastic global energy optimization procedure in Internal Coordinate Mechanics v. 3.7–2 (MolSoft, San Diego, CA, USA) [94,95] was used to build models of RBD_2_, RBD_3_ and RBD_4_. The 3.0 Å crystal structure of RBD_1_ of RanBP2 (PDB code 1RRP) was used as a modelling template, which has the following sequence identity with the query sequence: 59 per cent (RBD_2_), 51 per cent (RBD_3_) and 56 per cent (RBD_4_). The models were refined by globally optimizing the side chains and annealing the backbone using the biased-probability Monte Carlo method [94].

### 5.3. Cell culture and transient transfections

Mouse NIH/3T3 embryo fibroblasts (ATCC CRL-1658) were grown in Dulbecco's modified Eagle medium (DMEM, Gibco) supplemented with 10 per cent bovine calf serum and 1 per cent antibiotics (Invitrogen) at 37°C in presence of 5 per cent CO_2_. Upon 70 per cent confluency, cells were plated in 35 mm MatTek glass-bottomed culture dishes (MatTek Co.) for live-cell imaging and tracking studies. After 8 h of platting, the cells were transfected with 1 µg of YFP-KBD, YFP-KBD^*^, YFP-R_2_KR_3_, YFP-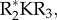 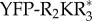 or 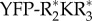 constructs cloned in mammalian expression vector pDest-733Y, using Fugene 6 or X-treamGENE 9 (Roche). KBD^*^ is KBD-Mut1 [24]. Transfection efficiencies for KBD and R_2_KR_3_ constructs (and mutant constructs thereof) were 20 per cent and 10 per cent, respectively. Immediately upon expression of KBD constructs (8, 10 or 12 h of post-transfection) and R_2_KR_3_ constructs (12 and 15 h of post-transfection), mitochondria of live cells were visualized upon incubation with MitoTracker Red CMXRos (Molecular Probes, no. M-7512) at 19 nM concentration for 30 min at 37°C. The growth medium was replaced by DMEM without serum just prior to live-cell imaging and time-lapse microscopy.

### 5.4. Time-lapse microscopy and image acquisitions

For live-cell imaging microscopy, culture dishes with transfected and stained mitochondria cells were placed into an integrated LiveCell System Chamber (Pathology Devices) in a controlled environment of 100 per cent humidity, 5 per cent CO_2_ and at 37°C. The live cell chamber was placed on a Nikon TE2000U microscope stage with a heated Plan Apochromat 60× objective lens (NA of 1.40). Images were acquired for 100 ms at 500 ms intervals for 1 minute (total of 121 fpm) by epifluoresence microscopy with Metamorph v. 6.3 software (Molecular Devices). Photobleaching and phototoxicity were prevented by shuttering the illumination fluorescence pathway with a filter wheel, and applying low exposure and reduced total recording times. Images were captured at non-saturating integration levels and 14-bit mono black/white with a high-quantum efficiency and sensitive CCD camera (Cascade 512K; Roper Scientific) mounted on a Nikon TE2000U microscope (Tokyo, Japan) equipped with appropriate excitation and emission filter wheels (Sutter Instruments, Novato, CA), 120 W mercury light source and an encoded motorized Z-Stage (Prior Scientific).

### 5.5. Mitochondrial tracking

Images for each dataset were collected from multiple cells in 6 to 12 culture dishes. Each mitochondrion trajectory was tracked and defined with the multi-dimensional, single-particle tracking and motion analysis modules of Metamorph v. 7.7.2.0. In line with other studies, the nucleus was used as a fixed reference point to establish mitochondrial directionality with the anterograde and retrograde motilities pointing away and towards the nucleus. *x–y* coordinates of a centroid were determined for each mitochondrion at every time point from time stacks composed of 121 frames. Briefly, mitochondrion tracking was aborted when (i) centroid intensity decreased by 40 per cent from the initial frame, (ii) fusion or collision between mitochondria occurred to exclude fusion and fission mitochondrial events, and (iii) the tracked mitochondria disappears from any frame during its trajectory. *x–y* coordinates of each mitochondrion trajectory were computed, and the data were automatically logged into Excel spreadsheets (Microsoft).

### 5.6. Data and statistical analyses

Motility profiles for each tracked mitochondrion were generated for each data group comprising approximately 100 mitochondria. All data points were analysed and processed for multiple biophysical parameters (see electronic supplementary material) with a customized batch-processing program developed by the Ferreira Laboratory using Origin v. 8.5 software (Northampton, MA), and that is available upon request. The five statistical parameters summarizing the datasets examined are provided in the electronic supplementary material (tables S1 and S2). Data for all the parameters examined were first tested for normality using the Shapiro–Wilk normality test (*α* = 0.05). Because none of the parameters examined were normally distributed (data not shown), the non-parametric Kruskal–Wallis test for group analysis and the Mann–Whitney *U-*test for two group comparisons were used at *α* = 0.001. Differences in medians were compared using Mood's median test at *α* = 0.01. The persistency of mitochondrial motility was assessed by chi-squared contingency test, whereas the degree of contingency was measured by Cramer's contingency coefficient (*ϕ*).

## 6. Acknowledgements

We thank Dr Alfred Wittinghofer for providing purified recombinant Ran GTPase charged with GppNHp, and Erin Haser and Tomas Moreno for the help with the pilot studies on mitochondrial motility. This work was supported by NIH grants GM083165, EY019492, 2P30-EY005722 and Research to Prevent Blindness to PAF. P.A.F. is the Jules & Doris Stein Research to Prevent Blindness Professor.

## Supplementary Material

Supplementary Data
