# Supplementary material for: Kinesin-1 and mitochondrial motility control by discrimination of structurally equivalent but distinct subdomains in Ran-GTP-binding domains of Ran-binding protein 2
Source: Open Biol. 2013 Mar;3(3):120183. doi: 10.1098/rsob.120183 (PMC3718338; doi:10.1098/rsob.120183)
Supplement: Supplementary Data [file rsob120183-s1.pdf]

SUPPLEMENTARY MATERIAL

Kinesin-1 and mitochondrial motility control by discrimination of structurally equivalent but distinct subdomains in Ran-GTP-binding domains of RanBP2

Hemangi Patil[1], Kyoung-in Cho[1], James Lee[1], Yi Yang[1] Andrew Orry[2] and Paulo A Ferreira[1,3, *]
[1] Department of Ophthalmology, Duke University Medical Center, Durham, NC 27710
[2]Molsoft, LLC; San Diego, CA 92121
[3]Department of Pathology, Duke University Medical Center, Durham, NC 27710

* To whom correspondence should be addressed: Paulo A. Ferreira, Duke University Medical Center, DUEC 3802, 2351 Erwin Road, Durham, NC 27710, USA; Tel. (919)-684-8457, Fax: (919)-684-3826; E-mail: paulo.ferreira@duke.edu

Supplementary Materials and Methods

Biophysical parameters definitions:

1. Motile event; motility of a mitochondrion in anterograde or retrograde direction with a velocity > 300 µm/sec between any two consecutive time frames.
2. Stationary event; stationary mitochondrion or motility of a mitochondrion of < 300 µm/sec in either direction between any two or more consecutive time frames.
3. Absolute duration of pause; time duration of stationary event between two motile events of a mitochondrion between a minimum of two consecutive time frames.
4. Frequency of change of direction; number of times a motile mitochondrion changes from anterograde to retrograde direction or vice versa during a total of 121 frames (60 seconds).
5. Persistency of motility; duration of a continuous (non-stop) motile event of a mitochondrion between two or more time frames.
6. Average velocity; the total trajectory distance traveled by a mitochondrion divided by its trajectory time during a total of 121 frames (excluding pauses).
7. Absolute velocity; the trajectory distance of a single non-stop run of a mitochondrion divided by its trajectory time.
8. Total displacement; the total distance traveled by a motile mitochondrion during 121 frames (60 seconds).

Mock KBD* KBD

(a)

Stationary Events (%)			
	8 hr	10 hr	12 hr
**KBD**	76.85*	66.11*	49.58*
**KBD***	86.77	85.95	81.81
**Mock**	90.08	86.77	84.29

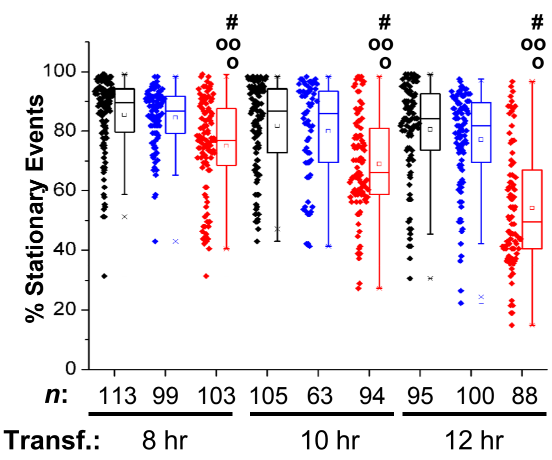

(b)

Total Anterograde Displacement (μm/min)			
	8 hr	10 hr	12 hr
**KBD**	1.53*	1.95*	3.74*
**KBD***	0.72	0.96	1.08
**Mock**	0.53	0.87	1.01

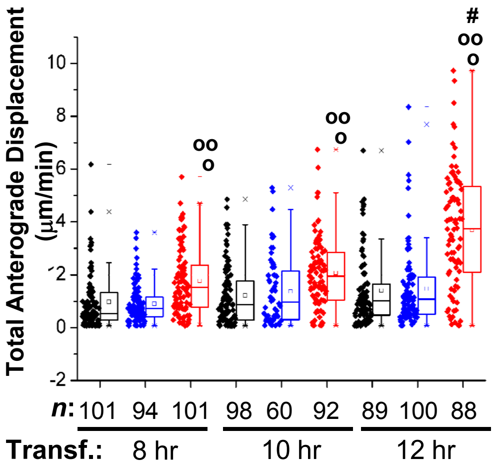

(c)

Total Retrograde Displacement (μm)			
	8 hr	10 hr	12 hr
**KBD**	1.48*	2.36*	4.08*
**KBD***	0.97	1.09	1.31
**Mock**	0.58	0.85	1.02

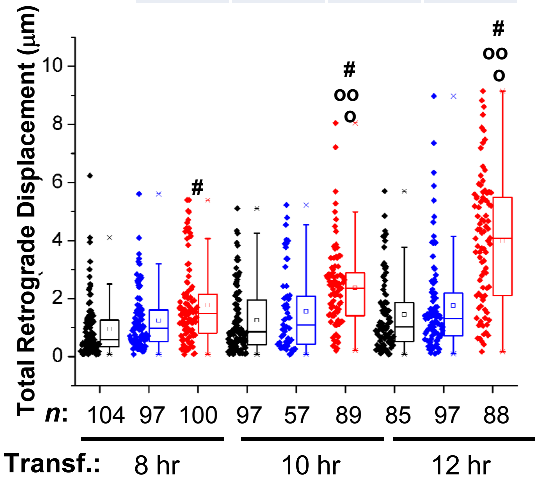

(d)

Total Displacement (μm)			
	8 hr	10 hr	12 hr
**KBD**	2.96*	4.28*	8.18*
**KBD***	1.75	1.79	2.22
**Mock**	1.11	1.48	1.9

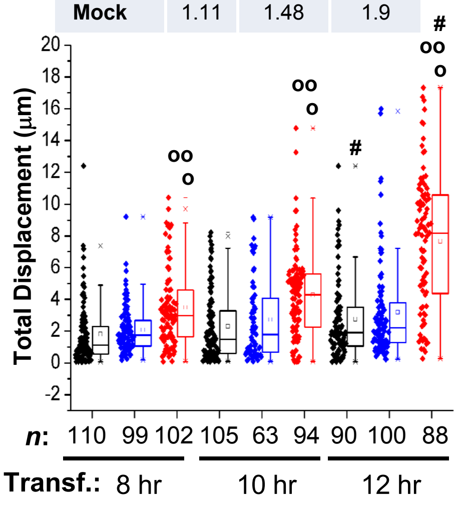

(e)

Frequency of Anterograde Motility (events/min)			
	8 hr	10 hr	12 hr
**KBD**	14*	17*	29*
**KBD***	6.5	9	10
**Mock**	6	8.5	9

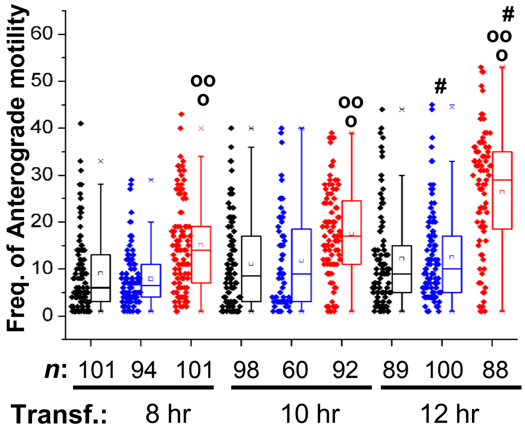

(f)

Frequency of Retrograde Motility (events/min)			
	8 hr	10 hr	12 hr
**KBD**	13*	22*	30.5*
**KBD***	8	10	12
**Mock**	6	8	9

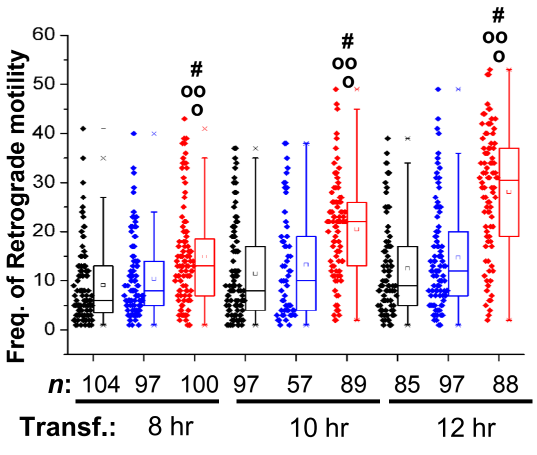

Supplementary Figure S1

Mock KBD* KBD

(g)

Absolute R-R Pause (sec)			
	8 hr	10 hr	12 hr
KBD	1.5*	1*	0.5*
KBD*	2	1.5	1.5
Mock	2.5	1.5	1.5

(h)

Absolute Duration of Pause (sec)			
	8 hr	10 hr	12 hr
KBD	1.5*	1*	1*
KBD*	2	1.5	1.5
Mock	2	1.5	1.5

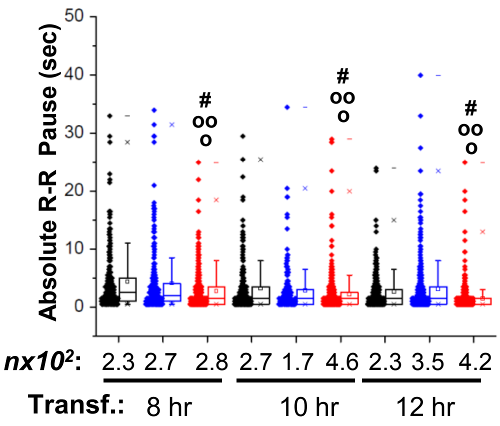

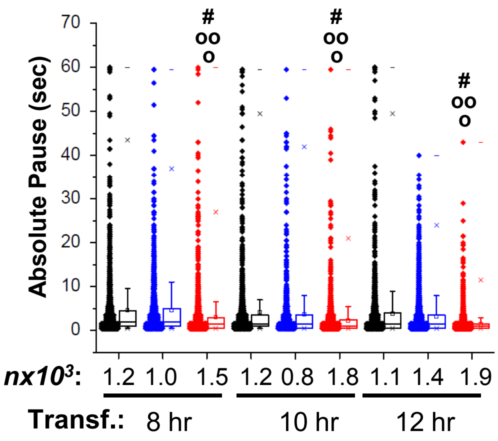

(i) Persistency of Retrograde Motility

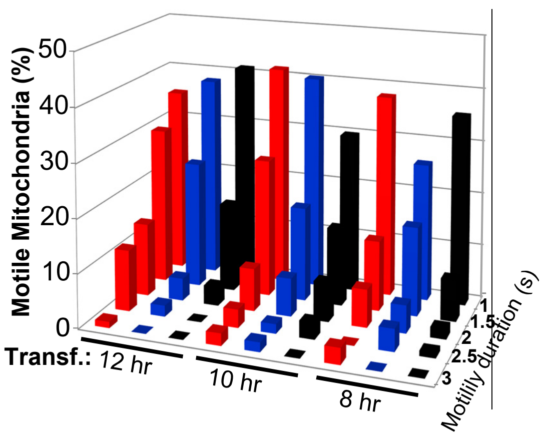

(j) Total Motility Persistency

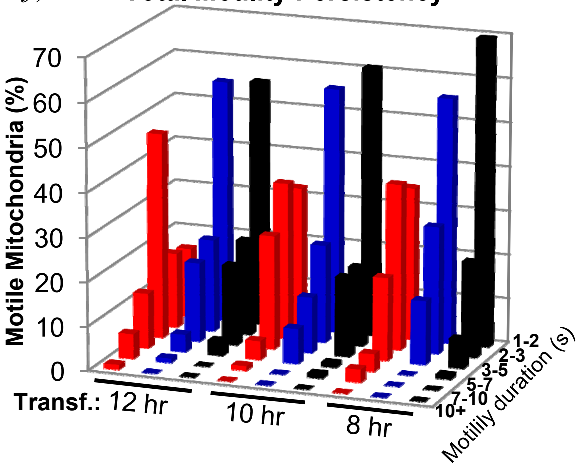

(k)

Average Velocity (μm/sec)			
	8 hr	10 hr	12 hr
KBD	0.45	0.43	0.50*
KBD*	0.46*	0.44*	0.42
Mock	0.38	0.40	0.43

(l)

Average Retrograde Velocity (μm/sec)			
	8 hr	10 hr	12 hr
KBD	0.44	0.43	0.52*
KBD*	0.46*	0.44*	0.43
Mock	0.37	0.41	0.44

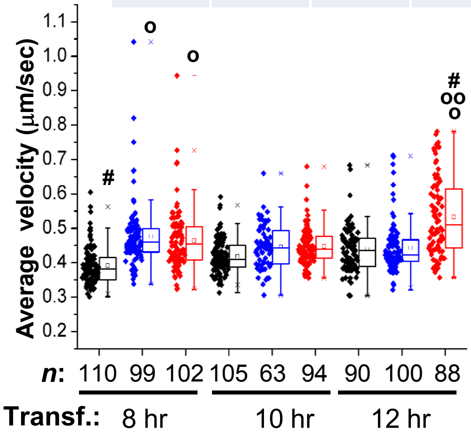

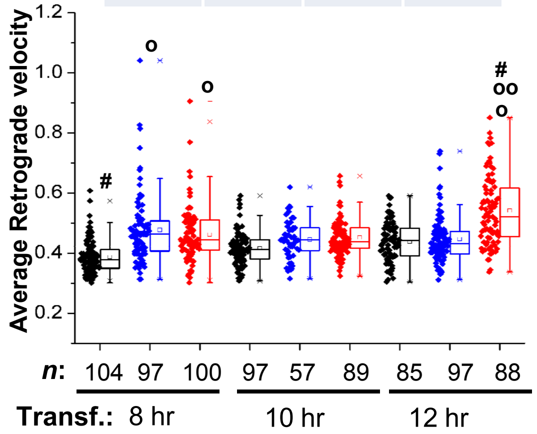

Supplementary Figure S1 (cont.)

Mock R_2*KR_3* R_2KR_3 R_2KR_3* R_2*KR_3

(*a*)

Stationary Events (%)		
	12 hr	15 hr
R_2KR_3	92.56	87.6
$R_2^*KR_3^*$	92.56	81.81
$R_2^*KR_3$	90.08	84.29
$R_2KR_3^*$	90.08	89.25
Mock	84.29*	66.94*

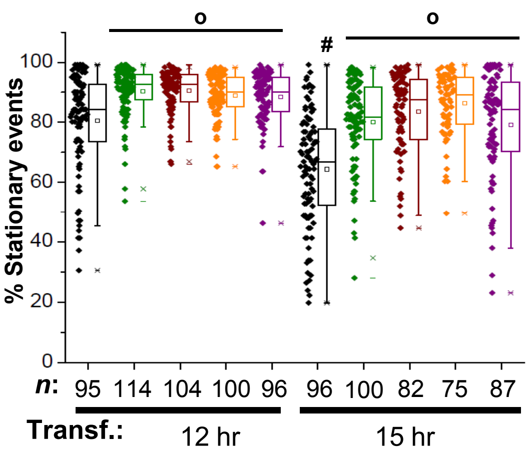

(*b*)

Total Anterograde Displacement (µm)		
	12 hr	15 hr
R_2KR_3	0.47	0.79
$R_2^*KR_3^*$	0.58	1.27
$R_2^*KR_3$	0.48	1.11
$R_2KR_3^*$	0.61	0.97
Mock	1.01*	2.29*

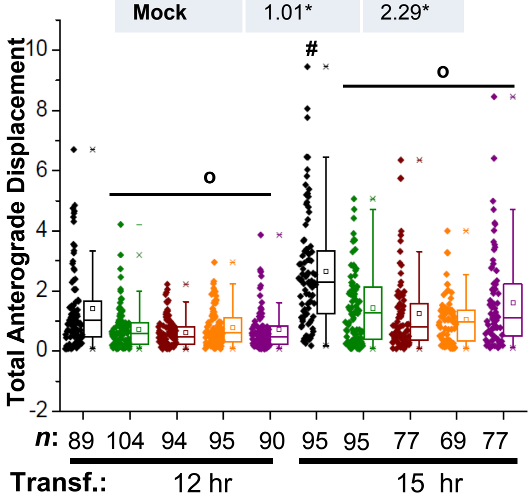

(*c*)

Total Retrograde Displacement (µm)		
	12 hr	15 hr
R_2KR_3	0.59	0.83
$R_2^*KR_3^*$	0.46	0.99
$R_2^*KR_3$	0.65	1.15
$R_2KR_3^*$	0.71	0.64*
Mock	1.02*	2.09*

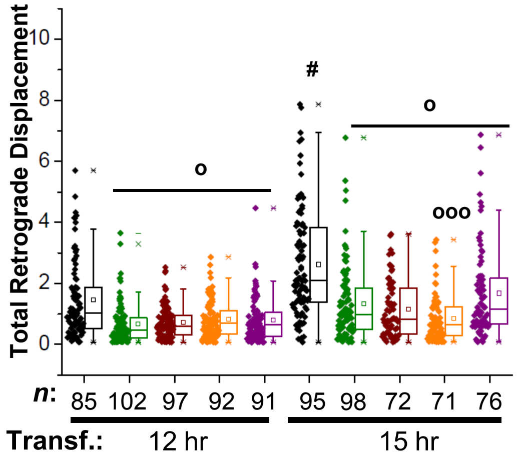

(*d*)

Total Displacement (µm)		
	12 hr	15 hr
R_2KR_3	0.97	1.53
$R_2^*KR_3^*$	0.93	2.26
$R_2^*KR_3$	1.12	2.06
$R_2KR_3^*$	1.27	1.38
Mock	1.90*	4.14*

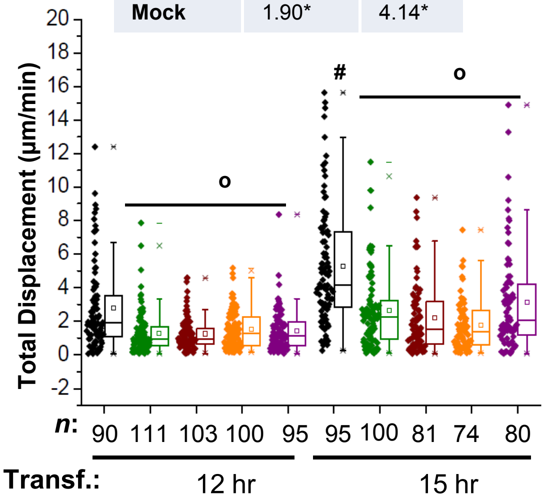

(*e*)

Frequency of Anterograde Motility (events/min)		
	12 hr	15 hr
R_2KR_3	4	7
$R_2^*KR_3^*$	5	12
$R_2^*KR_3$	5	10
$R_2KR_3^*$	5	9
Mock	9*	21*

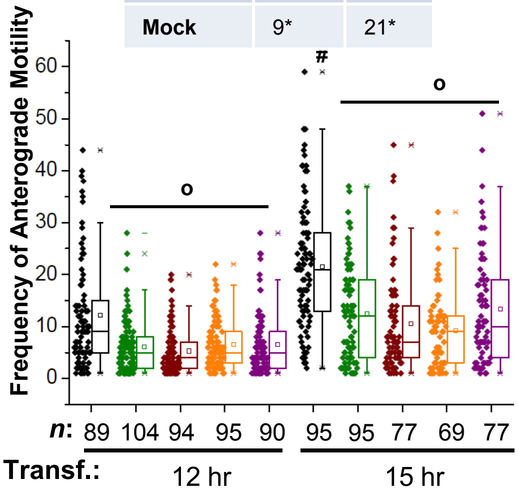

(*f*)

Frequency of Retrograde Motility (events/min)		
	12 hr	15 hr
R_2KR_3	5	8
$R_2^*KR_3^*$	4	10
$R_2^*KR_3$	6	11.5
$R_2KR_3^*$	5.5	6*
Mock	9*	19*

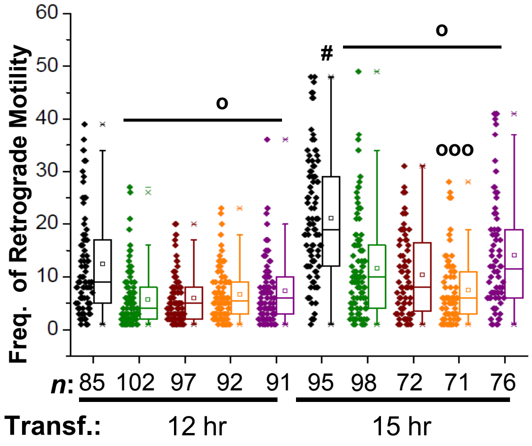

Supplementary Figure S2

(*g*)

Absolute R-R Pause (sec)		
	12 hr	15 hr
R_2KR_3	3	2
$R_2^*KR_3^*$	3	2
$R_2^*KR_3$	2.5	1.5
$R_2KR_3^*$	3	2
Mock	1.5*	1*

(*h*)

Absolute Duration of Pause (sec)		
	12 hr	15 hr
R_2KR_3	3.5	1.5
$R_2^*KR_3^*$	3.5	1.5
$R_2^*KR_3$	2.5	1.5
$R_2KR_3^*$	3	2
Mock	1.5*	1*

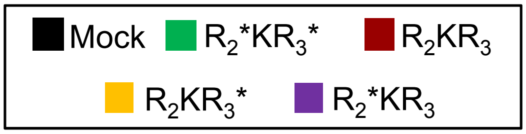

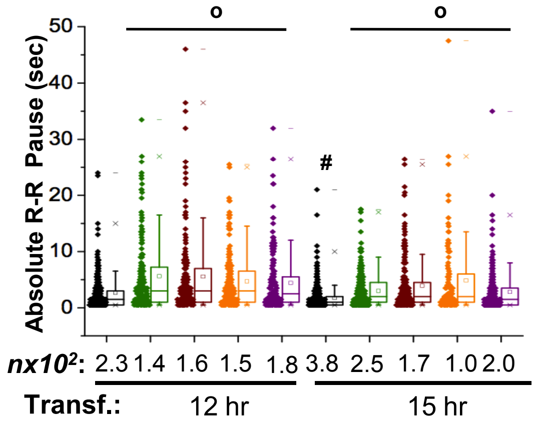

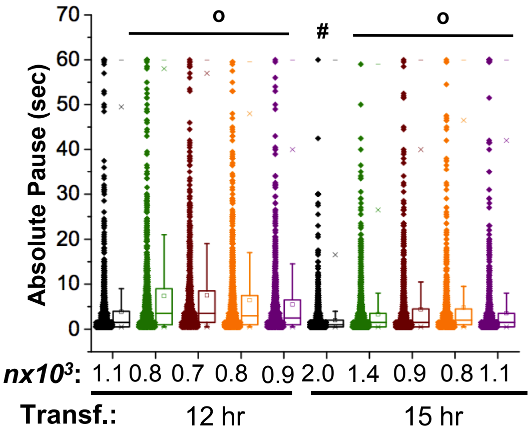

(*i*) **Persistency of Retrograde Motility**

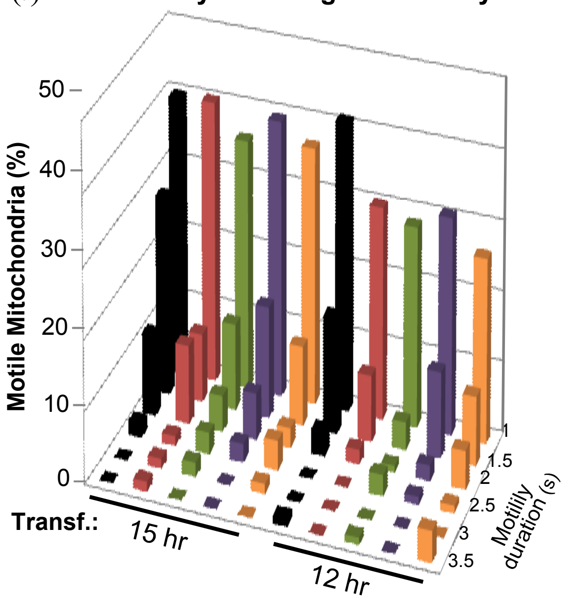

(*j*) **Total Motility Persistency**

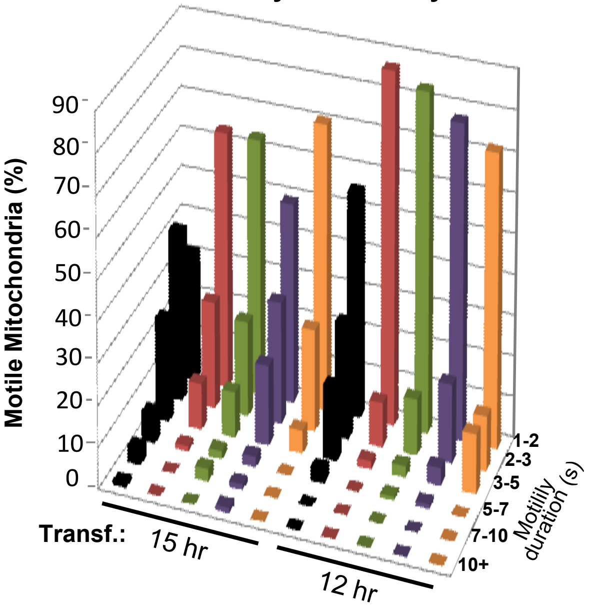

(*k*)

Average Velocity (μm/sec)		
	12 hr	15 hr
R_2KR_3	0.47*	0.41*
$R_2^*KR_3^*$	0.42	0.41*
$R_2^*KR_3$	0.41	0.43
$R_2KR_3^*$	0.48*	0.42
Mock	0.43	0.44

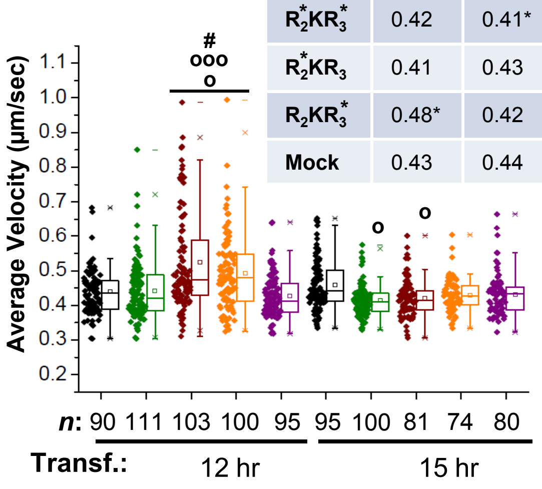

(*l*)

Average Retrograde Velocity (μm/sec)		
	12 hr	15 hr
R_2KR_3	0.46*	0.40
$R_2^*KR_3^*$	0.39	0.41
$R_2^*KR_3^*$	0.40	0.42
$R_2KR_3^*$	0.48*	0.42
Mock	0.44	0.44

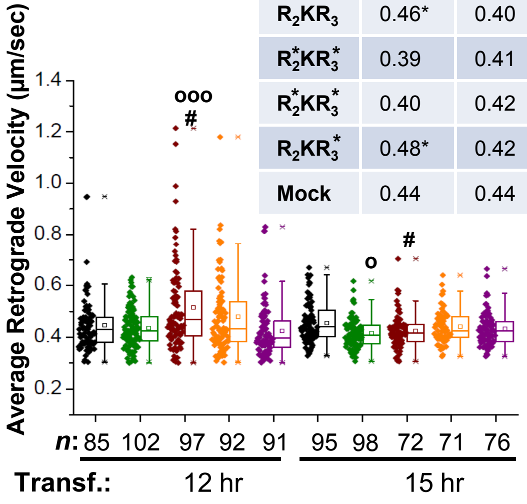

Supplementary Figure S2 (cont.)

Supplementary Figure Legends

Supplementary Figure S1. Up-regulation of biophysical parameters of mitochondrial kinetics by KBD of RanBP2. Dot-box plot analyses are shown in (*a*-*h*) and (*k*-*l*). The tables accompanying dot-box plots represent the corresponding median values for the represented data sets. KBD compared to KBD* and mock transfected cells causes temporally a decrease of stationary events (*a*), an increase of total anterograde (*b*) and retrograde displacements (*c*), an increase in total displacement (*d*), an increase in frequency of anterograde (*e*) and retrograde motilities (*f*), a decrease in absolute pauses between retrograde (*g*) and any motility events (*h*), an increase in average (*k*) and retrograde velocities (*l*) of mitochondria at 12 hours post-transfection. (*i*) 3D-histogram showing that an increase in the persistency of mitochondrial retrograde motility is contingent upon KBD, but not KBD^* or mock-transfected cells (chi square contingency test, 0.001<P <0.01, χ^2 = 23.77, Cramer's contingency coefficient ϕ =0.46), and upon the time of post-transfection (chi square contingency test, p<0.05, χ^2 = 18.76, Cramer's contingency coefficient ϕ =0.40); (*j*) 3D-histogram showing that an increase in the persistency of total mitochondrial motility is contingent upon KBD, but not KBD^* or mock-transfected cells (chi square contingency test, p<0.001, χ^2 = 63.15, Cramer's contingency coefficient ϕ =0.68) and upon the time of post-transfection (chi square contingency test, p<0.001, χ^2 = 42.09, Cramer's contingency coefficient ϕ =0.54). Legends: *, median significantly different (Mood's median test, α=0.01); ^{o}, ^{oo} and $^\#$ are mitochondrial pools significantly different from non-transfected (^{o}), KBD^* (^{oo}) and other times of transfection with the same construct ($^\#$), Mann Whitney test, p<0.0001; *n*, number of mitochondria; Mock, mock-transfected; KBD and KBD^* are wild-type and mutant kinesin-binding domain of RanBP2, respectively. *a*-*h* and *k*-*l*, Mann-Whitney test, *P*<0.001.

Supplementary Figure S2. Down-regulation and stereochemical selectivity of multiple biophysical parameters of mitochondrial motility by wild-type and mutant R_2KR_3 constructs of RanBP2. Dot-box plot analyses are shown in *a*-*h* and *k*-*l*. The tables accompanying dot-box plots represent the corresponding median values for the represented data sets. Wild-type R_2KR_3 and mutant constructs thereof with the

W→R mutation in the WKER motif of RBD_2 ($R^*_2KR_3$), RBD_3 ($R_2KR^*_3$) or both ($R^*_2KR^*_3$) compared with mock transfected cells cause temporally an increase of stationary events (*a*), a decrease of total anterograde (*b*)and retrograde displacements (*c*), a decrease in total displacement (*d*), a decrease in frequency of anterograde (*e*) and retrograde motilities (*f*), an increase in absolute pauses between retrograde (*g*) and any motility events (*h*), a decrease in retrograde (*k*) and average velocities (*l*) of mitochondria selectively in R_2KR_3 and $R_2KR^*_3$ transfected cells at 12 hours post-transfection. (*i*) 3D-histogram showing that the persistency of retrograde motility is neither contingent upon any R_2KR_3 constructs (chi square contingency test, $P>0.05$) nor upon the time of post-transfection (chi square contingency test, $P>0.05$). (*j*) A reduction in persistency of mitochondrial motility is significantly contingent upon transfection of any upon R_2KR_3 construct (chi square contingency test, $p<0.001$, $\chi^2 = 63.49$, Cramer's contingency coefficient $\phi = 0.32$). The persistency of mitochondrial motility of mock-transfected cells is contingent upon time of post-transfection (chi square contingency test, $p<0.05$, $\chi^2 = 14.99$, Cramer's contingency coefficient $\phi = 0.28$). Persistency of mitochondrial motility of any RKR construct is not contingent upon time of post-transfection (chi square contingency test, $p>0.05$). Legends: *, median significantly different (Mood's median test, $\alpha=0.01$); ^{o}, ^{ooo} and $^{\#}$ are mitochondrial pools significantly different from non-transfected (^{o}), R_2KR_3 constructs (^{ooo}) and other times of transfection with the same construct ($^{\#}$), Mann Whitney test, $p<0.0001$; *n*, number of mitochondria; Mock, mock-transfected. (*a*-*h*) and (*k*-*l*)

Movie S1. 3T3 cell transfected with YFP-KBD for 12 hours and stained with MitoTracker is shown. Images were analyzed by time-lapse and epifluorescence microscopy. Frames were taken every 0.5 sec for 1 min. Still image of Movie S1 is shown in figure 2*a* of the main text.

Movie S1*a*. Region of interest of a 3T3 cell shown in Movie S1 (lower panel of figure 2*a* of the main text) transfected with YFP-KBD for 12 hours and stained with MitoTracker. Images were analyzed by time-lapse and epifluorescence microscopy. Frames were taken every 0.5 sec for 1 min.

Movie S2. 3T3 cell transfected with YFP-R_2KR_3 for 15 hours and stained with MitoTracker is shown. Images were analyzed by time-lapse and epifluorescence microscopy. Frames were taken every 0.5 sec for 1 min. Still image of Movie S2 is shown in figure 2*b* of the main text.

Movie S2*a*. Region of interest of a 3T3 cell shown in Movie S2 (lower panel of figure 2*b* of the main text) transfected with YFP-R_2KR_3 for 15 hours and stained with MitoTracker. Images were analyzed by time-lapse and epifluorescence microscopy. Frames were taken every 0.5 sec for 1 min.

Movie S3. 3T3 cell mock transfected after 15 hours and stained with MitoTracker is shown. Images were analyzed by time-lapse and epifluorescence microscopy. Frames were taken every 0.5 sec for 1 min. Still image of Movie S3 is shown in figure 2*c* of the main text.

Movie S3*a*. Region of interest of a 3T3 cell shown in Movie S3 (lower panel of figure 2*c* of the main text) mock transfected after 15 hours and stained with MitoTracker. Images were analyzed by time-lapse and epifluorescence microscopy. Frames were taken every 0.5 sec for 1 min.

Supplementary Table 1. Summary of calculated biophysical parameters of mitochondrial motility illustrating data sample size (*n*, number of mitochondria), mode, and five number data summary (minimum, 1st quartile, median, 3rd quartile, maximum) for each data set. Legend: MOCK, mock-transfected; KBD, kinesin-binding domain; *, W→R mutation; R_2KR_3, RBD_2-KBD-RBD_3.

Hours of transfection and construct	*n* total	Mode	Minimum	1st Quartile (Q1)	Median	3rd Quartile (Q3)	Maximum
**A. AVERAGE VELOCITY (μm/second)**							
8 hr MOCK	110	--	0.30098	0.35045	0.38204	0.41502	0.60456
8 hr KBD*	99	0.37831	0.33793	0.43091	0.46022	0.49946	1.04127
8 hr KBD	102	0.41999	0.32291	0.40855	0.4536	0.50519	0.94317
10 hr MOCK	105	--	0.31255	0.38702	0.40913	0.44976	0.59135
10 hr KBD*	63	--	0.30542	0.39619	0.44384	0.49365	0.65958
10 hr KBD	94	--	0.35554	0.41314	0.43886	0.47728	0.67927
12 hr MOCK	90	--	0.30392	0.38908	0.43496	0.47051	0.68305
12 hr KBD*	100	--	0.32075	0.40403	0.42173	0.46631	0.71175
12 hr KBD	88	--	0.35672	0.44238	0.50928	0.615	0.78083
12 hr R_2KR_3	103	--	0.31064	0.42929	0.47508	0.59369	0.98693
12 hr $R^*_2KR^*_3$	111	--	0.30384	0.38561	0.42034	0.48795	0.85052
12 hr $R^*_2KR_3$	95	0.4631	0.31825	0.38148	0.41495	0.4631	0.64094
12 hr $R_2KR^*_3$	100	--	0.32476	0.41228	0.48036	0.54798	0.99421
15 hr MOCK	95	0.47607	0.33494	0.41261	0.44156	0.50142	0.65283
15 hr R_2KR_3	81	0.38728	0.30654	0.38728	0.41411	0.44266	0.60108
15 hr $R^*_2KR^*_3$	100	0.42224	0.3288	0.38195	0.41015	0.43493	0.57441
15 hr $R^*_2KR_3$	80	0.39172	0.32303	0.38693	0.43282	0.45338	0.66466
15 hr $R_2KR^*_3$	74	--	0.33414	0.40229	0.42721	0.45678	0.60394
**B. AVERAGE ANTEROGRADE VELOCITY (μm/second)**							
8 hr MOCK	101	--	0.3005	0.35766	0.39226	0.41339	0.64272
8 hr KBD*	94	0.33309	0.3048	0.39412	0.44512	0.50541	0.80555
8 hr KBD	101	0.40479	0.3125	0.40276	0.44765	0.50209	0.94317
10 hr MOCK	98	--	0.3006	0.38093	0.40552	0.44703	0.65975
10 hr KBD*	60	--	0.30542	0.37882	0.44582	0.48146	0.84154
10 hr KBD	92	0.41402	0.34756	0.41011	0.44259	0.47914	0.70808
12 hr MOCK	89	--	0.30392	0.37954	0.43095	0.47757	0.94736
12 hr KBD*	100	--	0.32425	0.38995	0.42761	0.46713	0.73983
12 hr KBD	88	--	0.3305	0.43197	0.50783	0.61227	0.8069
12 hr R_2KR_3	94	--	0.30099	0.40493	0.47031	0.59577	1.21396
12 hr $R^*_2KR^*_3$	104	--	0.30008	0.38496	0.42475	0.48087	0.63221
12 hr $R^*_2KR_3$	90	0.81351	0.30178	0.36757	0.40052	0.46301	0.82911
12 hr $R_2KR^*_3$	95	--	0.30276	0.38358	0.43279	0.53677	1.18009
15 hr MOCK	95	0.47357	0.32861	0.40239	0.44181	0.50406	0.66999

15 hr R_2KR_3	77	0.30916	0.30654	0.38365	0.41616	0.44962	0.70395
15 hr $R^*_2KR^*_3$	95	0.35272	0.30673	0.37466	0.4067	0.44702	0.61711
15 hr $R^*_2KR_3$	77	0.38691	0.32613	0.38407	0.42539	0.46138	0.66466
15 hr $R_2KR^*_3$	69	--	0.32614	0.39968	0.42495	0.47835	0.64019
**C. AVERAGE RETROGRADE VELOCITY (µm/second)**							
8 hr MOCK	104	--	0.30098	0.34967	0.37774	0.41151	0.60791
8 hr KBD*	97	0.3977	0.31371	0.40703	0.46291	0.50721	1.04127
8 hr KBD	100	0.42551	0.30158	0.41056	0.4436	0.51054	0.90561
10 hr MOCK	97	--	0.30784	0.38056	0.41366	0.4455	0.59135
10 hr KBD*	57	--	0.31552	0.40941	0.44335	0.48397	0.61971
10 hr KBD	89	--	0.32432	0.41631	0.43724	0.4843	0.65692
12 hr MOCK	85	--	0.30467	0.39132	0.44243	0.48217	0.59101
12 hr KBD*	97	--	0.31149	0.39793	0.43217	0.47286	0.73961
12 hr KBD	88	--	0.33748	0.45435	0.52128	0.61599	0.85101
12 hr R_2KR_3	97	--	0.31064	0.40441	0.46154	0.58637	1.65926
12 hr $R^*_2KR^*_3$	102	--	0.30667	0.36477	0.39817	0.47551	0.99201
12 hr $R^*_2KR_3$	91	0.30054	0.30054	0.37669	0.40388	0.455	0.86872
12 hr $R_2KR^*_3$	92	--	0.30777	0.39439	0.48402	0.58648	1.22352
15 hr MOCK	95	0.47819	0.30568	0.40991	0.44774	0.50869	0.69459
15 hr R_2KR_3	72	0.41852	0.31464	0.37894	0.40223	0.45256	0.66022
15 hr $R^*_2KR^*_3$	98	0.45342	0.30039	0.37667	0.41389	0.44617	0.57101
15 hr $R^*_2KR_3$	76	0.37877	0.31207	0.37946	0.42311	0.47243	0.64488
15 hr $R_2KR^*_3$	71	--	0.31642	0.36967	0.42057	0.44289	0.55928
**D. TOTAL DISPLACEMENT (µm/minute)**							
8 hr MOCK	110	--	0.07524	0.5653	1.11619	2.30556	12.40762
8 hr KBD*	99	0.95751	0.1868	1.05133	1.75083	2.68391	9.20704
8 hr KBD	102	1.57331	0.08042	1.62949	2.96463	4.5701	10.41825
10 hr MOCK	105	--	0.08569	0.59819	1.48524	3.28255	8.22977
10 hr KBD*	63	--	0.09447	0.67022	1.79086	4.0749	9.18009
10 hr KBD	94	--	0.08889	2.2524	4.28929	5.59389	14.78431
12 hr MOCK	90	--	0.07583	1.06404	1.90675	3.49333	12.40051
12 hr KBD*	100	--	0.23501	1.27599	2.22036	3.77591	15.99208
12 hr KBD	88	--	0.26992	4.37104	8.18931	10.57407	17.32344
12 hr R_2KR_3	103	--	0.08563	0.65646	0.97331	1.55557	4.58827
12 hr $R^*_2KR^*_3$	111	--	0.07991	0.55426	0.9365	1.68944	7.85724
12 hr $R^*_2KR_3$	95	0.57007	0.08275	0.56833	1.12408	1.96666	8.34378
12 hr $R_2KR^*_3$	100	--	0.16024	0.54333	1.27414	2.25106	5.17794
15 hr MOCK	95	7.27676	0.25518	2.83236	4.1434	7.30006	15.63323
15 hr R_2KR_3	81	0.70464	0.07893	0.64088	1.53149	3.15825	9.35999

15 hr $R*_2KR*_3$	100	1.86694	0.09838	0.95007	2.26198	3.22558	11.49638
15 hr $R*_2KR_3$	80	1.51445	0.08415	1.18722	2.06891	4.22255	14.90669
15 hr R_2KR*_3	74	--	0.10592	0.60685	1.38297	2.63177	7.42819
**E. TOTAL ANTEROGRADE DISPLACEMENT (µm/minute)**							
8 hr MOCK	101	--	0.07512	0.2857	0.53184	1.34224	6.17187
8 hr KBD*	94	0.25276	0.07838	0.42837	0.72632	1.15722	3.59736
8 hr KBD	101	0.40464	0.07812	0.7805	1.53118	2.3539	5.70283
10 hr MOCK	98	--	0.075	0.28498	0.87158	1.77579	4.8536
10 hr KBD*	60	--	0.08525	0.31096	0.9655	2.13483	5.28789
10 hr KBD	92	2.38427	0.08672	1.05075	1.95443	2.84807	6.73434
12 hr MOCK	89	--	0.07583	0.4748	1.01899	1.65811	6.6961
12 hr KBD*	100	--	0.08351	0.51316	1.08408	1.92832	8.34959
12 hr KBD	88	--	0.08326	2.07966	3.74258	5.33465	9.72095
12 hr R_2KR_3	94	--	0.07254	0.22982	0.47947	0.80985	2.22357
12 hr $R*_2KR*_3$	104	--	0.07991	0.22465	0.58618	0.93964	4.21307
12 hr $R*_2KR_3$	90	0.19484	0.07545	0.21363	0.48276	0.86224	3.86596
12 hr R_2KR*_3	95	--	0.07569	0.31428	0.61284	1.11451	2.94905
15 hr MOCK	95	3.3274	0.17877	1.23419	2.29923	3.3274	9.45156
15 hr R_2KR_3	77	0.16061	0.07893	0.36468	0.79201	1.58021	6.35033
15 hr $R*_2KR*_3$	95	0.27065	0.07975	0.37653	1.27131	2.12556	5.06549
15 hr $R*_2KR_3$	77	0.29724	0.10504	0.49598	1.11587	2.2281	8.44761
15 hr R_2KR*_3	69	--	0.08579	0.35124	0.97217	1.36476	4.00133
**F. TOTAL RETROGRADE DISPLACEMENT (µm/minute)**							
8 hr MOCK	104	--	0.07524	0.33802	0.58905	1.25362	6.23575
8 hr KBD*	97	0.70475	0.07843	0.51483	0.97509	1.60556	5.60968
8 hr KBD	100	1.16867	0.07539	0.81242	1.48723	2.1394	5.39631
10 hr MOCK	97	--	0.08173	0.41054	0.85599	1.9533	5.11233
10 hr KBD*	57	--	0.07888	0.43034	1.09488	2.08466	5.22529
10 hr KBD	89	--	0.20833	1.41352	2.36206	2.89618	8.04997
12 hr MOCK	85	--	0.07921	0.52563	1.02296	1.86372	5.70441
12 hr KBD*	97	--	0.0944	0.71309	1.31719	2.18355	8.97313
12 hr KBD	88	--	0.17112	2.09946	4.08435	5.50113	9.14756
12 hr R_2KR_3	97	--	0.0786	0.33916	0.59177	0.94594	2.52474
12 hr $R*_2KR*_3$	102	--	0.07698	0.20522	0.4674	0.87109	3.64417
12 hr $R*_2KR_3$	91	0.07498	0.07498	0.26123	0.65219	1.05509	4.47782
12 hr R_2KR*_3	92	--	0.07694	0.3453	0.71092	1.1157	2.85864
15 hr MOCK	95	3.94936	0.07642	1.37252	2.09014	3.83167	7.87046
15 hr R_2KR_3	72	0.54403	0.07866	0.35193	0.83531	1.83709	3.61473
15 hr $R*_2KR*_3$	98	2.11404	0.0778	0.48613	0.99045	1.84052	6.78031

15 hr R*$_2$KR$_3$	76	0.67693	0.08415	0.66395	1.15628	2.17111	6.87211
15 hr R$_2$KR*$_3$	71	--	0.08496	0.28433	0.64554	1.22291	3.42686
**G. FREQUENCY OF DIRECTION CHANGE (minute^{-1})**							
8 hr MOCK	101	1	1	3	6	15	44
8 hr KBD*	94	4	1	4	6	10	37
8 hr KBD	100	5	1	7	16	24	52
10 hr MOCK	95	2	1	3	9	21	39
10 hr KBD*	60	1	1	3	6.5	20	39
10 hr KBD	91	1	1	12	22	28	51
12 hr MOCK	86	1	1	5	9	16	47
12 hr KBD*	99	2	1	5	11	22	49
12 hr KBD	88	41	2	21	34	40.5	60
12 hr R$_2$KR$_3$	93	1	1	1	3	6	30
12 hr R*$_2$KR*$_3$	104	1	1	2	3	6	44
12 hr R*$_2$KR$_3$	89	2	1	3	6	9	29
12 hr R$_2$KR*$_3$	90	7	1	3	6	8	20
15 hr MOCK	95	18	2	14	23	32	60
15 hr R$_2$KR$_3$	77	1	1	3	6	14	34
15 hr R*$_2$KR*$_3$	99	2	1	4	9	15	43
15 hr R*$_2$KR$_3$	77	7	1	6	10	20	49
15 hr R$_2$KR*$_3$	70	2	1	2	6	10	34
**H. FREQUENCY OF STATIONARY MITOCHONDRIA (minute^{-1})**							
8 hr MOCK	113	112	38	97	109	114	120
8 hr KBD*	99	103	52	96	105	111	119
8 hr KBD	103	86	38	83	93	106	120
10 hr MOCK	105	118	52	88	105	114	119
10 hr KBD*	63	113	50	84	104	113	119
10 hr KBD	94	85	33	71	80	98	119
12 hr MOCK	95	102	37	89	102	112	120
12 hr KBD*	100	106	27	84	99	108.5	118
12 hr KBD	88	49	18	49	60	81	117
12 hr R$_2$KR$_3$	104	117	80	105.5	112	116	120
12 hr R*$_2$KR*$_3$	114	113	65	106	112	116	120
12 hr R*$_2$KR$_3$	96	117	56	101	109	115	120
12 hr R$_2$KR*$_3$	100	115	79	103	109	115	119
15 hr MOCK	96	59	24	63.5	81	94	120
15 hr R$_2$KR$_3$	82	108	54	90	106	114	120
15 hr R*$_2$KR*$_3$	100	95	34	90	99	111	119
15 hr R*$_2$KR$_3$	87	120	28	85	102	113	120

15 hr $R_2KR^*_3$	75	116	60	96	108	115	120
**I. FREQUENCY OF ANTEROGRADE MITOCHONDRIA MOTILITY ($minute^{-1}$)**							
8 hr MOCK	101	1	1	3	6	13	41
8 hr KBD*	94	4	1	4	6.5	11	29
8 hr KBD	101	19	1	7	14	19	43
10 hr MOCK	98	1	1	3	8.5	17	40
10 hr KBD*	60	3	1	3	9	18.5	40
10 hr KBD	92	11	1	11	17	24.5	39
12 hr MOCK	89	5	1	5	9	15	44
12 hr KBD*	100	4	1	5	10	17	45
12 hr KBD	88	32	1	18.5	29	35	53
12 hr R_2KR_3	94	2	1	2	4	7	20
12 hr $R^*_2KR^*_3$	104	1	1	2	5	8	28
12 hr $R^*_2KR_3$	90	2	1	2	5	9	28
12 hr $R_2KR^*_3$	95	2	1	3	5	9	22
15 hr MOCK	95	17	2	13	21	28	59
15 hr R_2KR_3	77	4	1	4	7	14	45
15 hr $R^*_2KR^*_3$	95	2	1	4	12	19	37
15 hr $R^*_2KR_3$	77	3	1	4	10	19	51
15 hr $R_2KR^*_3$	69	1	1	3	9	12	32
**J. FREQUENCY OF RETROGRADE MITOCHONDRIA MOTILITY ($minute^{-1}$)**							
8 hr MOCK	104	5	1	3.5	6	13	41
8 hr KBD*	97	3	1	5	8	14	40
8 hr KBD	100	3	1	7	13	18.5	43
10 hr MOCK	97	1	1	4	8	17	37
10 hr KBD*	57	3	1	4	10	19	38
10 hr KBD	89	22	2	13	22	26	49
12 hr MOCK	85	9	1	5	9	17	39
12 hr KBD*	97	8	1	7	12	20	49
12 hr KBD	88	31	2	19	30.5	37	53
12 hr R_2KR_3	97	2	1	2	5	8	20
12 hr $R^*_2KR^*_3$	102	2	1	2	4	8	27
12 hr $R^*_2KR_3$	91	2	1	3	6	10	36
12 hr $R_2KR^*_3$	92	3	1	3	5.5	9	23
15 hr MOCK	95	18	1	12	19	29	48
15 hr R_2KR_3	72	3	1	3.5	8	16.5	31
15 hr $R^*_2KR^*_3$	98	2	1	4	10	16	49
15 hr $R^*_2KR_3$	76	7	1	6	11.5	19	41
15 hr $R_2KR^*_3$	71	6	1	3	6	11	28

**K. PERSISTENCY OF MITOCHONDRIA MOTILITY (Seconds)**							
8 hr MOCK	106	1	1	1	1.5	2.5	5.5
8 hr KBD*	97	2	1	1.5	2	3	4.5
8 hr KBD	101	2.5	1	2	2.5	3.5	11.5
10 hr MOCK	100	1.5	1	1.5	2	3	7.5
10 hr KBD*	62	1.5	1	1.5	2	3	6.5
10 hr KBD	93	2.5	1	2	2.5	3.5	9.5
12 hr MOCK	88	1.5	1	1.5	2	3	6.5
12 hr KBD*	100	1.5	1	1.5	2	3	7.5
12 hr KBD	88	3.5	1	3	3.5	4.5	14.5
12 hr R_2KR_3	101	1	1	1	1.5	2	4
12 hr $R*_2KR*_3$	110	1	1	1	1.5	2	6.5
12 hr $R*_2KR_3$	94	1.5	1	1	1.5	2	5.5
12 hr R_2KR*_3	97	1.5	1	1	1.5	2.5	4.5
15 hr MOCK	95	2	1	2	3	4	12.5
15 hr R_2KR_3	80	1.5	1	1.5	2	2.5	9
15 hr $R*_2KR*_3$	99	2	1	1.5	2	2.5	8.5
15 hr $R*_2KR_3$	77	1.5	1	1.5	2	3	10.5
15 hr R_2KR*_3	72	1.5	1	1.5	1.5	2.5	5
**L. PERSISTENCY OF RETROGRADE MITOCHONDRIA MOTILITY (Seconds)**							
8 hr MOCK	48	1	1	1	1	1	2.5
8 hr KBD*	50	1	1	1	1.25	1.5	2.5
8 hr KBD	61	1	1	1	1	1.5	3
10 hr MOCK	54	1	1	1	1	1.5	3
10 hr KBD*	39	1	1	1	1	1.5	3
10 hr KBD	73	1	1	1	1	1.5	3
12 hr MOCK	53	1	1	1	1	1.5	3.5
12 hr KBD*	66	1	1	1	1	1.5	2.5
12 hr KBD	81	1	1	1	1.5	2	4
12 hr R_2KR_3	40	1	1	1	1	1.5	2
12 hr $R*_2KR*_3$	38	1	1	1	1	1	3.5
12 hr $R*_2KR_3$	43	1	1	1	1	1.5	4
12 hr R_2KR*_3	44	1	1	1	1	1.75	4
15 hr MOCK	78	1	1	1	1.5	1.5	2.5
15 hr R_2KR_3	47	1	1	1	1	1.5	3.5
15 hr $R*_2KR*_3$	57	1	1	1	1	1.5	3
15 hr $R*_2KR_3$	50	1	1	1	1	1.5	4.5
15 hr R_2KR*_3	40	1	1	1	1	1.5	3

**M. PERSISTENCY OF AMOCKEROGRADE MITOCHONDRIA MOTILITY (Seconds)**							
8 hr MOCK	45	1	1	1	1	1.5	3
8 hr KBD*	49	1	1	1	1	1.5	2.5
8 hr KBD	64	1	1	1	1	1.5	3
10 hr MOCK	54	1	1	1	1	1.5	3
10 hr KBD*	33	1	1	1	1	1.5	2.5
10 hr KBD	73	1	1	1	1	1.5	2.5
12 hr MOCK	50	1	1	1	1	1.5	3
12 hr KBD*	56	1	1	1	1	1	2.5
12 hr KBD	73	1.5	1	1	1.5	2	3
12 hr R_2KR_3	23	1	1	1	1	1.5	2
12 hr $R^*_2KR^*_3$	45	1	1	1	1	1.5	5.5
12 hr $R^*_2KR_3$	27	1	1	1	1	1.5	5
12 hr $R_2KR^*_3$	39	1	1	1	1	1.5	3.5
15 hr MOCK	79	1	1	1	1	1.5	3
15 hr R_2KR_3	47	1	1	1	1.5	1.5	5.5
15 hr $R^*_2KR^*_3$	60	1	1	1	1.5	2	5
15 hr $R^*_2KR_3$	51	1	1	1	1	2	3
15 hr $R_2KR^*_3$	39	1	1	1	1	1.5	4.5
**N. MAXIMUM DURATION OF PAUSE (Seconds)**							
8 hr MOCK	113	11.5	1.5	9	18.5	29.5	60
8 hr KBD*	99	9.5	2.5	12	17	27	59.5
8 hr KBD	103	3.5	2	6.5	12	25	60
10 hr MOCK	105	5.5	2.5	7.5	16.5	31	59.5
10 hr KBD*	63	5.5	2.5	7	15.5	30	59.5
10 hr KBD	94	4.5	2	4.5	6.5	11.5	59.5
12 hr MOCK	95	10	1.5	8.5	13.5	28.5	60
12 hr KBD*	100	8	1.5	8	13.75	21	40
12 hr KBD	88	2.5	1	2.5	3.5	8.5	43
12 hr R_2KR_3	104	23	5	17.25	25	36.5	60
12 hr $R^*_2KR^*_3$	114	15.5	5	16	23.75	34.5	60
12 hr $R^*_2KR_3$	96	11	2.5	12.5	19	28.75	60
12 hr $R_2KR^*_3$	100	19	5.5	14.5	21.25	33	59.5
15 hr MOCK	96	4	1	4	6.5	10.25	60
15 hr R_2KR_3	82	6	3	11	17	27	60
15 hr $R^*_2KR^*_3$	100	10.5	2	8	12	19	59
15 hr $R^*_2KR_3$	87	60	1.5	7	13	27	60
15 hr $R_2KR^*_3$	75	13.5	4.5	11.5	17.5	32	60

**O. % MOTILE MITOCHONDRIA**							
8 hr MOCK	113	0.82645	0	4.95868	9.09091	19.00826	67.7686
8 hr KBD*	99	7.43802	0.82645	7.43802	12.39669	19.83471	56.19835
8 hr KBD	103	4.13223	0	11.57025	22.31405	30.57851	67.7686
10 hr MOCK	105	1.65289	0.82645	4.95868	12.39669	26.44628	56.19835
10 hr KBD*	63	5.78512	0.82645	5.78512	13.22314	29.75207	57.85124
10 hr KBD	94	28.92562	0.82645	18.18182	33.05785	40.49587	71.90083
12 hr MOCK	95	14.87603	0	6.61157	14.87603	25.61983	68.59504
12 hr KBD*	100	11.57025	1.65289	9.50413	17.35537	29.75207	76.8595
12 hr KBD	88	58.67769	2.47934	32.2314	49.58678	58.67769	84.29752
12 hr R_2KR_3	104	2.47934	0	3.30579	6.61157	11.98347	33.05785
12 hr $R^*_2KR^*_3$	114	1.65289	0	3.30579	6.61157	11.57025	45.45455
12 hr $R^*_2KR_3$	96	2.47934	0	4.13223	9.09091	15.70248	52.89256
12 hr $R_2KR^*_3$	100	4.13223	0.82645	4.13223	9.09091	14.04959	33.8843
15 hr MOCK	96	22.31405	0	21.4876	32.2314	46.69421	79.33884
15 hr R_2KR_3	82	4.13223	0	4.95868	11.57025	24.79339	54.54545
15 hr $R^*_2KR^*_3$	100	2.47934	0.82645	7.43802	17.35537	24.79339	71.07438
15 hr $R^*_2KR_3$	87	0	0	5.78512	14.87603	28.92562	76.03306
15 hr $R_2KR^*_3$	75	3.30579	0	4.13223	9.91736	19.83471	49.58678
**P. % STATIONARY MITOCHONDRIA**							
8 hr MOCK	113	92.56198	31.40496	80.16529	90.08264	94.21488	99.17355
8 hr KBD*	99	85.12397	42.97521	79.33884	86.77686	91.73554	98.34711
8 hr KBD	103	71.07438	31.40496	68.59504	76.8595	87.60331	99.17355
10 hr MOCK	105	97.52066	42.97521	72.72727	86.77686	94.21488	98.34711
10 hr KBD*	63	93.38843	41.32231	69.42149	85.95041	93.38843	98.34711
10 hr KBD	94	70.24793	27.27273	58.67769	66.1157	80.99174	98.34711
12 hr MOCK	95	84.29752	30.57851	73.55372	84.29752	92.56198	99.17355
12 hr KBD*	100	87.60331	22.31405	69.42149	81.81818	89.66942	97.52066
12 hr KBD	88	40.49587	14.87603	40.49587	49.58678	66.94215	96.69421
12 hr R_2KR_3	104	96.69421	66.1157	87.19008	92.56198	95.86777	99.17355
12 hr $R^*_2KR^*_3$	114	93.38843	53.71901	87.60331	92.56198	95.86777	99.17355
12 hr $R^*_2KR_3$	96	96.69421	46.28099	83.47107	90.08264	95.04132	99.17355
12 hr $R_2KR^*_3$	100	95.04132	65.28926	85.12397	90.08264	95.04132	98.34711
15 hr MOCK	96	48.76033	19.83471	52.47934	66.94215	77.68595	99.17355
15 hr R_2KR_3	82	89.2562	44.6281	74.38017	87.60331	94.21488	99.17355
15 hr $R^*_2KR^*_3$	100	78.5124	28.09917	74.38017	81.81818	91.73554	98.34711
15 hr $R^*_2KR_3$	87	99.17355	23.1405	70.24793	84.29752	93.38843	99.17355
15 hr $R_2KR^*_3$	75	95.86777	49.58678	79.33884	89.2562	95.04132	99.17355

Supplementary Table 2. Summary of calculated biophysical parameters of mitochondrial motility illustrating data sample size (*n*, number of events), mode, and five number data summary (minimum, 1st quartile, median, 3rd quartile, maximum) for each data set. Legend: MOCK, mock-transfected; KBD, kinesin-binding domain; *, W→R mutation; R_2KR_3, RBD_2-KBD-RBD_3.

Hours of transfection and construct	*n* total	Mode	Minimum	1st Quartile (Q1)	Median	3rd Quartile (Q3)	Maximum
**A. ABSOLUTE RETROGRADE VELOCITY (μm/second)**							
8 hr MOCK	945	--	0.30014	0.32956	0.3766	0.45648	1.42412
8 hr KBD*	1014	0.30673	0.30009	0.34161	0.41293	0.52509	2.03607
8 hr KBD	1490	0.31894	0.30033	0.34198	0.42065	0.5369	1.63384
10 hr MOCK	1114	0.77282	0.30012	0.33776	0.39477	0.49111	1.19824
10 hr KBD*	755	--	0.30004	0.34333	0.41487	0.53418	1.75346
10 hr KBD	1900	0.72632	0.30008	0.34414	0.41216	0.52094	1.50841
12 hr MOCK	1059	--	0.3	0.34205	0.41184	0.52168	1.40046
12 hr KBD*	1422	0.96166	0.30019	0.34346	0.41825	0.55064	1.42365
12 hr KBD	2476	0.68	0.30011	0.37339	0.49506	0.68141	3.82691
12 hr R_2KR_3	578	--	0.30009	0.33268	0.39399	0.51459	2.07567
12 hr $R^*_2KR^*_3$	585	--	0.30039	0.33934	0.40232	0.51865	1.241
12 hr $R^*_2KR_3$	681	0.3	0.3	0.33345	0.38977	0.48754	1.28816
12 hr $R_2KR^*_3$	619	--	0.30021	0.34374	0.40535	0.56133	1.66425
15 hr MOCK	2205	0.96166	0.30006	0.36295	0.45726	0.63345	1.43795
15 hr R_2KR_3	744	0.30243	0.30021	0.33651	0.39334	0.49118	1.00894
15 hr $R^*_2KR^*_3$	1140	0.30117	0.3002	0.33673	0.39931	0.48669	1.13726
15 hr $R^*_2KR_3$	1070	0.30492	0.30015	0.34121	0.40709	0.52158	1.17867
15 hr $R_2KR^*_3$	535	0.308	0.30042	0.33452	0.39513	0.47948	1.40921
**B. ABSOLUTE ANTEROGRADE VELOCITY (μm/second)**							
8 hr MOCK	913	0.96166	0.3005	0.33421	0.38298	0.47495	1.32322
8 hr KBD*	738	0.31081	0.30009	0.33148	0.39831	0.51034	1.49296
8 hr KBD	1515	0.31413	0.30006	0.34196	0.41955	0.53304	1.7296
10 hr MOCK	1086	0.96166	0.30003	0.33488	0.3937	0.49566	1.19971
10 hr KBD*	708	--	0.30012	0.34729	0.40677	0.52514	1.41517
10 hr KBD	1698	0.9473	0.30012	0.34717	0.41793	0.52673	1.66921
12 hr MOCK	1081	--	0.30013	0.34008	0.40906	0.52238	1.767
12 hr KBD*	1240	0.68	0.30004	0.34354	0.42141	0.539	1.23785
12 hr KBD	2316	0.68	0.30002	0.37129	0.49404	0.68136	3.65459
12 hr R_2KR_3	494	--	0.30027	0.33199	0.38401	0.50964	2.09517
12 hr $R^*_2KR^*_3$	639	--	0.30008	0.33626	0.40165	0.51794	1.4481
12 hr $R^*_2KR_3$	599	0.30874	0.30021	0.33406	0.38394	0.49029	1.18274
12 hr $R_2KR^*_3$	632	--	0.30001	0.33834	0.40528	0.5319	1.70893
15 hr MOCK	2251	0.96166	0.30008	0.36044	0.45483	0.62517	1.45388

15 hr R_2KR_3	811	0.30779	0.30022	0.34276	0.4141	0.52203	1.37807
15 hr $R^*_2KR^*_3$	1181	0.32303	0.30005	0.33891	0.3957	0.49266	1.17488
15 hr $R^*_2KR_3$	1027	0.30051	0.30015	0.34526	0.41412	0.53774	1.1918
15 hr $R_2KR^*_3$	633	0.30713	0.30007	0.33853	0.39843	0.50558	1.03573
C. **ABSOLUTE VELOCITY (μm/second)**							
8 hr MOCK	1858	0.96166	0.30014	0.33192	0.38026	0.46579	1.42412
8 hr KBD*	1752	0.30673	0.30009	0.33807	0.4052	0.52023	2.03607
8 hr KBD	3005	0.68	0.30006	0.34198	0.41998	0.53645	1.7296
10 hr MOCK	2200	0.77282	0.30003	0.33649	0.39439	0.49428	1.19971
10 hr KBD*	1463	--	0.30004	0.34464	0.41083	0.53065	1.75346
10 hr KBD	3598	0.72632	0.30008	0.34593	0.41522	0.52544	1.66921
12 hr MOCK	2140	--	0.3	0.34084	0.41017	0.52213	1.767
12 hr KBD*	2662	0.96166	0.30004	0.34354	0.42051	0.54435	1.42365
12 hr KBD	4792	0.68	0.30002	0.37221	0.49454	0.68136	3.82691
12 hr R_2KR_3	1072	--	0.30009	0.33204	0.3891	0.51225	2.09517
12 hr $R^*_2KR^*_3$	1224	--	0.30008	0.338	0.402	0.51858	1.4481
12 hr $R^*_2KR_3$	1280	0.3	0.3	0.33348	0.38682	0.48773	1.28816
12 hr $R_2KR^*_3$	1251	--	0.30001	0.34196	0.40535	0.54399	1.70893
15 hr MOCK	4456	0.96166	0.30006	0.36127	0.45611	0.62963	1.45388
15 hr R_2KR_3	1555	0.30243	0.30021	0.33997	0.40285	0.50583	1.37807
15 hr $R^*_2KR^*_3$	2321	0.30117	0.30005	0.33775	0.39705	0.49023	1.17488
15 hr $R^*_2KR_3$	2097	0.30051	0.30015	0.34366	0.41104	0.5298	1.1918
15 hr $R_2KR^*_3$	1168	0.30713	0.30007	0.33648	0.39692	0.49353	1.40921
D. **DURATION OF PAUSE (A-A) (Seconds)**							
8 hr MOCK	210	0.5	0.5	1	2	4.5	34
8 hr KBD*	163	0.5	0.5	1	3	6.5	44.5
8 hr KBD	324	0.5	0.5	0.5	1.5	3	31.5
10 hr MOCK	254	0.5	0.5	1	1.5	3.5	49.5
10 hr KBD*	163	0.5	0.5	1	1.5	3.5	27
10 hr KBD	354	0.5	0.5	0.5	1	2	15
12 hr MOCK	276	0.5	0.5	0.5	1.5	3.5	37.5
12 hr KBD*	263	0.5	0.5	0.5	1.5	3	27
12 hr KBD	396	0.5	0.5	0.5	1	1.5	19.5
12 hr R_2KR_3	140	0.5	0.5	1.25	3	6.5	44.5
12 hr $R^*_2KR^*_3$	144	0.5	0.5	1.25	3	7.25	39
12 hr $R^*_2KR_3$	121	1.5	0.5	1.5	2.5	5	31.5
12 hr $R_2KR^*_3$	175	0.5	0.5	1	3	6	38
15 hr MOCK	438	0.5	0.5	0.5	1	2	12
15 hr R_2KR_3	176	0.5	0.5	1	1.75	4.5	34
15 hr $R^*_2KR^*_3$	276	0.5	0.5	0.5	1.5	4	29

15 hr $R^*_2KR_3$	201	0.5	0.5	0.5	1.5	3	31
15 hr $R_2KR^*_3$	166	0.5	0.5	1	3	6	32
E. **DURATION OF PAUSE (R-R) (Seconds)**							
8 hr MOCK	233	1	0.5	1	2.5	5	33
8 hr KBD*	278	0.5	0.5	1	2	4	51.5
8 hr KBD	282	0.5	0.5	0.5	1.5	3.5	25
10 hr MOCK	270	0.5	0.5	0.5	1.5	3.5	58
10 hr KBD*	173	0.5	0.5	0.5	1.5	3	34.5
10 hr KBD	466	0.5	0.5	0.5	1	2.5	29
12 hr MOCK	232	0.5	0.5	0.5	1.5	3	24
12 hr KBD*	354	0.5	0.5	0.5	1.5	3.5	40
12 hr KBD	421	0.5	0.5	0.5	0.5	1.5	25
12 hr R_2KR_3	167	1	0.5	1	3	7	46
12 hr $R^*_2KR^*_3$	144	1	0.5	1	3	7.25	33.5
12 hr $R^*_2KR_3$	181	0.5	0.5	1	2.5	5.5	32
12 hr $R_2KR^*_3$	152	0.5	0.5	1	3	6.5	25.5
15 hr MOCK	387	0.5	0.5	0.5	1	2	21
15 hr R_2KR_3	179	0.5	0.5	1	2	4.5	26.5
15 hr $R^*_2KR^*_3$	250	0.5	0.5	1	2	4.5	17.5
15 hr $R^*_2KR_3$	203	0.5	0.5	0.5	1.5	3.5	35
15 hr $R_2KR^*_3$	101	0.5	0.5	1	2	6	47.5
F. **DURATION OF PAUSE (R-A) (Seconds)**							
8 hr MOCK	354	0.5	0.5	1	2	5.5	60
8 hr KBD*	231	0.5	0.5	1	2	6.5	59.5
8 hr KBD	375	0.5	0.5	0.5	1	3	60
10 hr MOCK	350	0.5	0.5	1	1.75	4.5	59.5
10 hr KBD*	235	0.5	0.5	0.5	1.5	4	59.5
10 hr KBD	495	0.5	0.5	0.5	1	2.5	59.5
12 hr MOCK	338	0.5	0.5	1	2	5.5	60
12 hr KBD*	424	0.5	0.5	0.5	1.5	4	34.5
12 hr KBD	465	0.5	0.5	0.5	1	1.5	43
12 hr R_2KR_3	245	0.5	0.5	1.5	4	11.5	60
12 hr $R^*_2KR^*_3$	262	0.5	0.5	1.5	4	11.5	60
12 hr $R^*_2KR_3$	247	1	0.5	1.5	4	9	60
12 hr $R_2KR^*_3$	238	1	0.5	1.5	3.5	13.5	59.5
15 hr MOCK	569	0.5	0.5	0.5	1	2	60
15 hr R_2KR_3	256	0.5	0.5	1	2	6.5	60
15 hr $R^*_2KR^*_3$	332	0.5	0.5	1	2	4	59
15 hr $R^*_2KR_3$	258	0.5	0.5	0.5	2	4.5	60
15 hr $R_2KR^*_3$	185	0.5	0.5	1	3	6.5	60

G. **DURATION OF PAUSE (A-R) (Seconds)**							
8 hr MOCK	369	0.5	0.5	1	2.5	6	60
8 hr KBD*	242	0.5	0.5	1	2.5	6.5	59.5
8 hr KBD	375	0.5	0.5	0.5	1.5	3.5	60
10 hr MOCK	351	0.5	0.5	1	2	4.5	59.5
10 hr KBD*	237	0.5	0.5	0.5	1.5	3.5	59.5
10 hr KBD	519	0.5	0.5	0.5	1	2.5	59.5
12 hr MOCK	328	0.5	0.5	0.75	2	6	60
12 hr KBD*	408	0.5	0.5	0.5	1.5	4	35.5
12 hr KBD	462	0.5	0.5	0.5	1	1.5	29
12 hr R_2KR_3	261	1	0.5	1.5	4.5	13.5	60
12 hr $R^*_2KR^*_3$	258	0.5	0.5	1.5	5	13	60
12 hr $R^*_2KR_3$	243	1	0.5	1.5	3.5	10	60
12 hr $R_2KR^*_3$	235	0.5	0.5	1	3	10.5	59.5
15 hr MOCK	560	0.5	0.5	0.5	1	2	60
15 hr R_2KR_3	247	0.5	0.5	0.5	2	5.5	60
15 hr $R^*_2KR^*_3$	325	0.5	0.5	1	2	6	59
15 hr $R^*_2KR_3$	266	0.5	0.5	1	2	4.5	60
15 hr $R_2KR^*_3$	189	0.5	0.5	1	2.5	8	60
H. **ABSOLUTE DURATION OF PAUSE (Seconds)**							
8 hr MOCK	1241	0.5	0.5	1	2	4.5	60
8 hr KBD*	1094	0.5	0.5	1	2	5	59.5
8 hr KBD	1553	0.5	0.5	0.5	1.5	3	60
10 hr MOCK	1276	0.5	0.5	1	1.5	3.5	59.5
10 hr KBD*	816	0.5	0.5	0.5	1.5	3.5	59.5
10 hr KBD	1897	0.5	0.5	0.5	1	2.5	59.5
12 hr MOCK	1197	0.5	0.5	0.5	1.5	4	60
12 hr KBD*	1463	0.5	0.5	0.5	1.5	3.5	40
12 hr KBD	1976	0.5	0.5	0.5	1	1.5	43
12 hr R_2KR_3	763	0.5	0.5	1.5	3.5	8.5	60
12 hr $R^*_2KR^*_3$	863	0.5	0.5	1	3.5	9	60
12 hr $R^*_2KR_3$	955	0.5	0.5	1	2.5	6.5	60
12 hr $R_2KR^*_3$	863	0.5	0.5	1	3	7.5	59.5
15 hr MOCK	2039	0.5	0.5	0.5	1	2	60
15 hr R_2KR_3	936	0.5	0.5	0.5	1.5	4.5	60
15 hr $R^*_2KR^*_3$	1445	0.5	0.5	0.5	1.5	3.5	59
15 hr $R^*_2KR_3$	1171	0.5	0.5	0.5	1.5	3.5	60
15 hr $R_2KR^*_3$	819	0.5	0.5	1	2	4.5	60

Supplementary Table 3. Summary of persistency of motility of mitochondria represented as percentage after normalizing for total motile events. (A, B) Anterograde and retrograde persistency of motility in cells transfected with KBD (A) or R_2KR_3 constructs (B). (C, D) Total persistency of mitochondria motility of cells transfected with KBD (C) or R_2KR_3 constructs (D). Legend: MOCK, mock-transfected; KBD, kinesin-binding domain; *, W→R mutation; R_2KR_3, RBD_2-KBD-RBD_3.

**Table 3A**	**Anterograde**					**Retrograde**				
**Persistence of motility (seconds)**	**1**	**1.5**	**2**	**2.5**	**3**	**1**	**1.5**	**2**	**2.5**	**3**
Hours of transfection and construct										
12 hr KBD	25	27.27	23.86	5.68	1.14	35.23	29.55	13.64	11.36	1.14
12 hr KBD*	44	7	4	1	0	38.14	23.71	4.12	2.06	0
12 hr MOCK	35.96	13.48	5.62	0	1.12	41.18	16.47	3.53	0	0
10 hr KBD	51.09	19.57	7.61	1.09	0	41.57	25.84	7.87	3.37	2.25
10 hr KBD*	28.33	13.33	11.67	1.67	0	40.35	17.54	7.02	1.75	1.75
10 hr MOCK	30.61	16.33	3.06	2.04	3.06	29.9	14.43	7.22	3.09	0
8 hr KBD	37.62	21.78	1.98	0	0.99	38	13	7	0	3
8 hr KBD*	30.85	14.89	4.26	2.13	0	25.77	16.49	5.15	4.12	0
8 hr MOCK	27.72	10.89	1.98	2.97	0	35.58	7.69	1.92	0.96	0

**Table 3B**	**Anterograde**						**Retrograde**					
**Persistence of motility (seconds)**	**1 s**	**1.5**	**2**	**2.5**	**3**	**3.5**	**1**	**1.5**	**2**	**2.5**	**3**	**3.5**
15 hr MOCK	44.21	22.11	11.58	2.11	3.16	0	40	28.42	11.58	2.11	0	0
15 hr R_2KR_3	24.68	22.08	5.19	2.6	1.3	1.3	40.28	9.72	11.11	1.39	1.39	1.39
15 hr $R^*_2KR^*_3$	25.26	20	13.68	2.11	1.05	0	35.71	12.24	5.1	3.06	2.04	0
15 hr $R^*_2KR_3$	37.66	11.69	10.39	5.19	1.3	0	39.47	15.79	6.58	2.63	0	0
15 hr $R_2KR^*_3$	33.33	11.59	8.7	1.45	0	0	36.62	11.27	2.82	4.23	1.41	0
12 hr MOCK	35.96	13.48	5.62	0	1.12	0	41.18	16.47	3.53	0	0	1.18
12 hr R_2KR_3	17.2	6.45	1.08	0	0	0	30.21	9.38	2.08	0	0	0
12 hr $R^*_2KR^*_3$	27.88	11.54	1.92	0.96	0	0	28.43	3.92	0.98	2.94	0	0.98
12 hr $R^*_2KR_3$	20	4.44	2.22	2.22	1.11	0	30.77	12.09	2.2	1.1	0	0
12 hr $R_2KR^*_3$	28.42	8.42	1.05	1.05	0	2.11	26.09	9.78	5.43	1.09	0	4.35

Table 3C.

Total Persistency of motility (seconds)						
**seconds**	**>10**	**7 - 10**	**5 - 7**	**3 - 5**	**2 - 3**	**2 - 1**
12 hr KBD	1.136	5.6818	12.5	46.59	17.045	15.91
12 hr KBD*	0	1	4	18	21	55
12 hr MOCK	0	0	3.41	18.18	21.591	55.68
10 hr KBD	0	1.0753	4.3	25.81	35.484	32.26
10 hr KBD*	0	0	7.94	12.7	22.222	55.56
10 hr MOCK	0	1	1	18	18	61
8 hr KBD	0	2.9703	3.96	18.81	37.624	34.65
8 hr KBD*	0	0	0	14.43	28.866	55.67
8 hr MOCK	0	0	0.94	6.604	21.698	69.81

Table 3D.

Total Persistency of motility (seconds)						
**seconds**	**>10**	**7 - 10**	**5 - 7**	**3 - 5**	**2 - 3**	**2 - 1**
15 hr MOCK	0	0	1.25	11.25	26.25	63.75
15 hr R_2KR_3	0	3.0303	2.02	11.11	23.232	63.64
15 hr $R^*_2KR^*_3$	1.299	1.2987	2.6	19.48	29.87	49.35
15 hr $R^*_2KR_3$	0	0	0	5.556	25	70.83
15 hr $R_2KR^*_3$	0	0	3.41	18.18	28.409	55.68
12 hr MOCK	0	0	0	1.98	10.891	87.13
12 hr R_2KR_3	0	0	0.91	2.727	13.636	83.64
12 hr $R^*_2KR^*_3$	0	0	1.06	4.255	19.149	77.66
12 hr $R^*_2KR_3$	0	0	0	14.43	13.402	72.16
12 hr $R_2KR^*_3$	1.053	4.2105	7.37	25.26	42.105	31.58
